# Auf zur Wasserstoffentwicklung: Das Infrarot‐Spektrum von hydratisiertem Aluminiumhydrid‐Hydroxid HAlOH^+^(H_2_O)_*n*−1_, *n=*9–14

**DOI:** 10.1002/ange.202105166

**Published:** 2021-06-04

**Authors:** Jakob Heller, Wai Kit Tang, Ethan M. Cunningham, Ephrem G. Demissie, Christian van der Linde, Wing Ka Lam, Milan Ončák, Chi‐Kit Siu, Martin K. Beyer

**Affiliations:** ^1^ Institut für Ionenphysik und Angewandte Physik Universität Innsbruck Technikerstraße 25 6020 Innsbruck Österreich; ^2^ Department of Chemistry City University of Hong Kong 83 Tat Chee Avenue Kowloon Tong Hong Kong SAR P. R. China

**Keywords:** Metallhydrid, Protonentransfer, Schwingungsspektroskopie, Wasserspaltung, Wasserstoffbrückengebundenes Netzwerk

## Abstract

Hydratisierte Al^+^‐Ionen eliminieren H_2_ in einem Bereich von 11 bis 24 Wassermolekülen. Wir untersuchten die Struktur von HAlOH^+^(H_2_O)_n−1_, n=9–14, durch IR‐Mehrfachphotonendissoziationsspektroskopie bei 1400–2250 cm^−1^. Aufgrund quantenchemischer Rechnungen ordnen wir die Merkmale bei 1940 und 1850 cm^−1^ der Al‐H‐Streckschwingung in fünf‐ bzw. sechsfach koordinierten Al^III^‐Komplexen zu. Es werden Wasserstoffbrücken in Richtung des Hydrids beobachtet, beginnend bei n=12. Die Frequenz der Al‐H‐Streckschwingung ist sehr empfindlich gegenüber der Struktur des Netzwerks aus Wasserstoffbrücken, und die große Anzahl von Isomeren führt zu einer deutlichen Verbreiterung und Rotverschiebung der Absorptionen der wasserstoffbrückengebundenen Al‐H‐Streckschwingung. Das Hydrid kann sogar als doppelter Wasserstoffbrücken‐Akzeptor wirken und die Al‐H‐Streckschwingung zu Frequenzen verschieben, die unter denen der Wasser‐Biegemoden liegen. Das Einsetzen der Wasserstoffbrückenbindung und das Verschwinden der freien Al‐H‐Streckschwingung fallen mit dem Einsetzen der Wasserstoffentwicklung zusammen.

## Graphical abstract

Wassermoleküle müssen sich auf eine bestimmte Art um eine Aluminiumhydrid‐Hydroxid‐Einheit anordnen, um die Abspaltung molekularen Wasserstoffs zu ermöglichen. IR‐Mehrfachphotonendissoziationsspektroskopie zufolge tritt die Wasserstoffbrücke zum Hydrid in einem sechsfach koordinierten Al^III^‐Hydrid‐Hydroxid‐Komplex auf. Die kleinste Clustergröße, bei der die Hydrid‐Wasserstoffbrücke dominiert, ist auch diejenige, bei der die Wasserstoffentwicklung effizient wird.

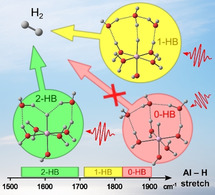

Die Wasserstoffentwicklung (hydrogen evolution reaction, HER) ist der Schlüssel zur Speicherung von überschüssiger erneuerbarer Energie durch Wasserelektrolyse^[1]^ sowie zur direkten Lichternte durch Photokatalysatoren.^[2]^ In elektrochemischen HER‐Studien wird üblicherweise die Netto‐Halbreaktion an der Kathode berichtet, bei der zwei Protonen mit zwei Elektronen rekombinieren und H_2_ bilden. Für den Reaktionsmechanismus sind zwei Wege denkbar: Die Wasserstoffentwicklung kann über die Rekombination von zwei an der Oberfläche adsorbierten Wasserstoffatomen^[3]^ oder über Hydrid‐Proton‐Rekombination erfolgen.^[4]^ Die Bildung des H_2_‐Moleküls aus zwei freien H‐Atomen ist energetisch anspruchsvoll und spielt in praktischen Prozessen keine Rolle. Die mechanistischen Details sind von höchster Relevanz für die Entwicklung neuartiger Elektrokatalysatoren und effizienter Elektrolyseure.

Hydratisierte Metallionen in der Gasphase sind wichtige Modellsysteme, um die Wasserstoffentwicklung auf molekularer Ebene zu untersuchen. In Gasphasenclustern zeigen mehrere Systeme Wasserstoffentwicklung unter dem Einfluss der Schwarzkörperstrahlung bei Raumtemperatur,^[5]^ insbesondere Mg^+^(H_2_O)_*n*_,^[6]^ Al^+^(H_2_O)_*n*_[^7^, ^8^] und V^+^(H_2_O)_*n*_.^[9]^ Die photochemische Wasserstoffbildung wurde auch für Mg^+^(H_2_O)_*n*_ ^[10]^ und V^+^(H_2_O)_*n*_ untersucht.^[11]^ Die Bildung von H_2_ aus Al^+^(H_2_O)_*n*_, die durch Schwarzkörperstrahlung aktiviert wird, zeigt eine interessante Größenabhängigkeit,[^7^, ^8^] die einige Hinweise auf mögliche Mechanismen gab. Quantenchemische Berechnungen von Reinhard und Niedner‐Schatteburg^[12]^ sowie Ab‐initio‐Moleküldynamik‐Simulationen von Siu und Liu^[13]^ zeigten, dass die Reaktion in zwei Schritten abläuft: Zunächst findet ein koordinierter Protonentransfer durch einen Wasser‐“Draht” aus mindestens drei H_2_O‐Molekülen statt, von einem Wassermolekül der ersten Schale auf die andere Seite des Al^+^‐Zentrums, wo das Proton zu Hydrid reduziert und gleichzeitig Al^I^ zu Al^III^ oxidiert wird. Dies führt zur Bildung eines hydratisierten Hydrid‐Hydroxid‐Komplexes, HAlOH^+^(H_2_O)_*n*−1_. Diese Insertionsreaktion wurde bereits 1995 von Watanabe und Iwata quantenchemisch modelliert.^[14]^ Ein zweiter Protonentransfer von einem Wassermolekül der ersten Schale, wiederum über einen Wasserdraht, der mit dem Hydrid verbunden ist, welches als Wasserstoffbrückenakzeptor dient, führt zur Bildung von H_2_ zusammen mit Al(OH)_2_^+^(H_2_O)_*n*−2_. Bisher ist der einzige indirekte experimentelle Nachweis für diesen Mechanismus ein H_2_O/D_2_O‐Austauschexperiment, das zeigt, dass der Protonentransfer in Al^+^(H_2_O)_*n*_ stattfindet,^[15]^ was das Vorhandensein der Hydrid‐Hydroxid‐Struktur HAlOH^+^(H_2_O)_*n*−1_ unterstützt. Es bleibt jedoch noch unklar, ob die Wasserstoffbrückenbindung hin zum Hydrid wirklich existiert und ob diese Struktureigenschaft über einen längeren Zeitraum stabil ist oder sofort zur H_2_‐Eliminierung führt.

Hier untersuchten wir die Spektroskopie von hydratisierten Aluminium‐Ionen Al^+^(H_2_O)_*n*_ in der Gasphase, *n=*9–14, mittels Infrarot‐Mehrfachphotonendissoziationsspektroskopie (IRMPD)^[16]^ im Bereich von 1400–2250 cm^−1^. Die Ionen wurden in einer Laserverdampfungsquelle^[17]^ erzeugt und in einer ICR‐Zelle gespeichert, die auf ca. 85 K gekühlt ist, um den Einfluss der Schwarzkörperstrahlung zu minimieren.^[18]^ Die Cluster‐Ionen wurden mit Licht aus einem durchstimmbaren optisch‐parametrischen Oszillatorsystem (OPO) bestrahlt, das mit einer Pulsfrequenz von 1000 Hz betrieben wurde, was einer quasi‐kontinuierlichen Bestrahlung auf der Zeitskala des ICR‐Experiments entspricht. Die zu untersuchende Clustergröße wurde durch resonante Anregung von unerwünschten Ionen massenselektiert, für 0,2 s bestrahlt, und es wurde ein Massenspektrum aufgenommen. Dieser Vorgang wurde für jede Infrarot‐Wellenzahl 15‐mal wiederholt, um das Signal‐zu‐Rauschen‐Verhältnis zu verbessern. Die Photonenabsorption führte zur Verdampfung von Wassermolekülen und in einigen Fällen zur H_2_‐Eliminierung. Die Intensität der Fragmente wurde mittels Massenspektrometrie quantifiziert. Typische Massenspektren werden in Abbildung S2 gezeigt.

Abbildung 1 zeigt die IRMPD‐Spektren für *n=*9–14. In diesem Größenbereich sagt die Theorie die HAlOH^+^(H_2_O)_*n*−1_‐Struktur voraus,[^12^, ^13^] und *n=*11 ist der kleinste Cluster, für den die H_2_‐Bildung berichtet wurde.[^7^, ^8^] Im *n=*9‐Spektrum wird die markante Bande bei 1610 cm^−1^ der Wasserbiegeschwingung zugeordnet, während die Bande bei 1940 cm^−1^ in der Nähe der *ν*_3_‐Mode (Al‐H‐Streckschwingung) von AlH_3_ liegt, die von Andrews und Mitarbeitern berichtet wurde.^[19]^ Dies bestätigt das Vorliegen der Hydrid‐Hydroxid‐Struktur. Bei *n=*10 tritt eine rotverschobene Bande um 1870 cm^−1^ auf, die auf die Koexistenz von zwei chemisch unterschiedlichen Aluminiumhydrid‐Spezies hinweist. Bei *n=*11 ist das Merkmal bei 1940 cm^−1^ fast verschwunden, und der Bereich der Wasser‐Biegemoden wird breiter. Bei *n=*12 setzt die H_2_‐Entwicklung ein, ausgelöst durch Infrarotstrahlung, und das verbleibende Merkmal im Aluminiumhydrid‐Streckbereich verschiebt sich zu 1850 cm^−1^. Der Verlust von H_2_+*x* H_2_O, *x=*2, 3 ist bei *n=*13 gleich intensiv wie der Verlust von H_2_O, und der Al‐H‐Streckbereich verbreitert sich beträchtlich und verschiebt sich ins Rote. Gleichzeitig verliert die Schulter zu höheren Energien im H_2_O‐Biegebereich an Intensität. Dieser Trend ist für *n=*14 stärker ausgeprägt, während die H_2_‐Entwicklung eine geringere Rolle spielt als für *n=*13. Die H_2_‐Bildung wird aufgrund der exothermen Reaktion von der Verdampfung von zwei bis drei Wassermolekülen begleitet, was sowohl mit den früheren BIRD‐Experimenten[^7^, ^8^] als auch mit der Theorie übereinstimmt.[^12^, ^13^]

**Figure 1:**
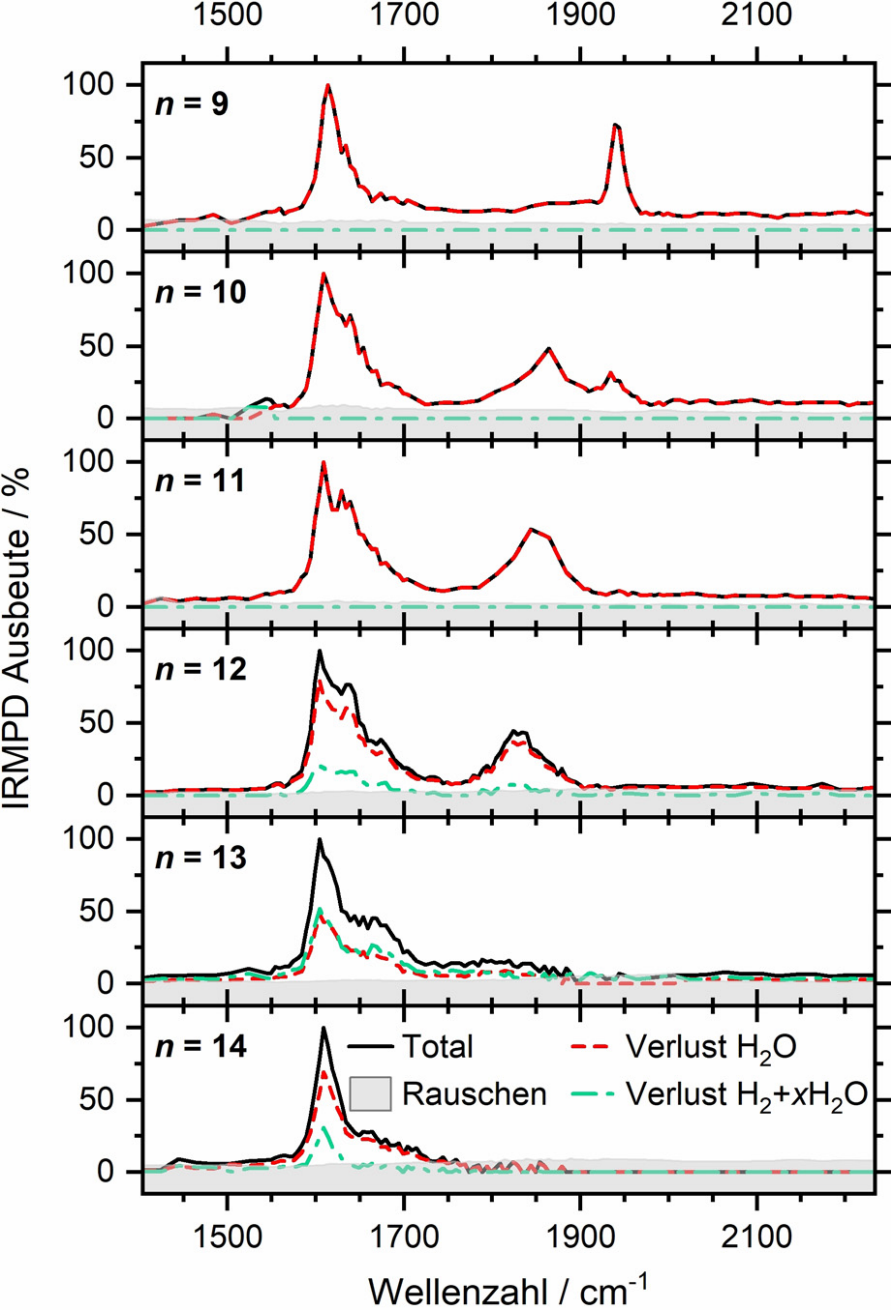
Experimentelle IRMPD‐Spektren von Al^+^(H_2_O)_*n*_, *n=*9–14.

Quantenchemische Berechnungen auf dem B3LYP/6‐311++G**‐Theorieniveau wurden mit dem Softwarepaket Gaussian durchgeführt.^[20]^ Die Infrarotspektren energetisch niedrig liegender Strukturen wurden durch Anwendung eines Skalierungsfaktors von 0,982 und einer Gaußschen Verbreiterung mit 20 cm^−1^ Halbwertsbreite auf die harmonischen Frequenzen simuliert.^[21]^ Die Energien wurden auf dem M06/6‐311++G**‐Theorieniveau nach erneuter Geometrieoptimierung ausgewertet; während das M06‐Funktional dazu neigt, unrealistische Abweichungen der Absorptionsintensitäten vorherzusagen,^[22]^ ist es dafür bekannt, dass es zuverlässigere Energien für wasserstoffgebundene Systeme liefert als B3LYP.^[23]^ Alle angegebenen Energien sind nullpunktkorrigiert und verwenden harmonische Frequenzen ohne Skalierung. Abbildung 2 zeigt die Strukturen mit der niedrigsten Energie, die wir bei unserer umfangreichen Suche gefunden haben; weitere Strukturen sind in den Hintergrundinformationen aufgeführt. Es wurden vier‐, fünf‐ und sechsfach koordinierte HAlOH^+^(H_2_O)_*n*−1_‐Komplexe untersucht, die mit ***n*** **‐4 c**, ***n*** **‐5 c** bzw. ***n*** **‐6 c** bezeichnet werden. Für *n*≥11 wird die Wasserstoffbrückenbindung zum Hydrid energetisch konkurrenzfähig, und Komplexe mit einer und zwei Wasserstoffbrückenbindungen zum Hydrid werden als ***n*** **‐6 c‐HB** bzw. ***n*** **‐6 c‐HB2** bezeichnet.

**Figure 2:**
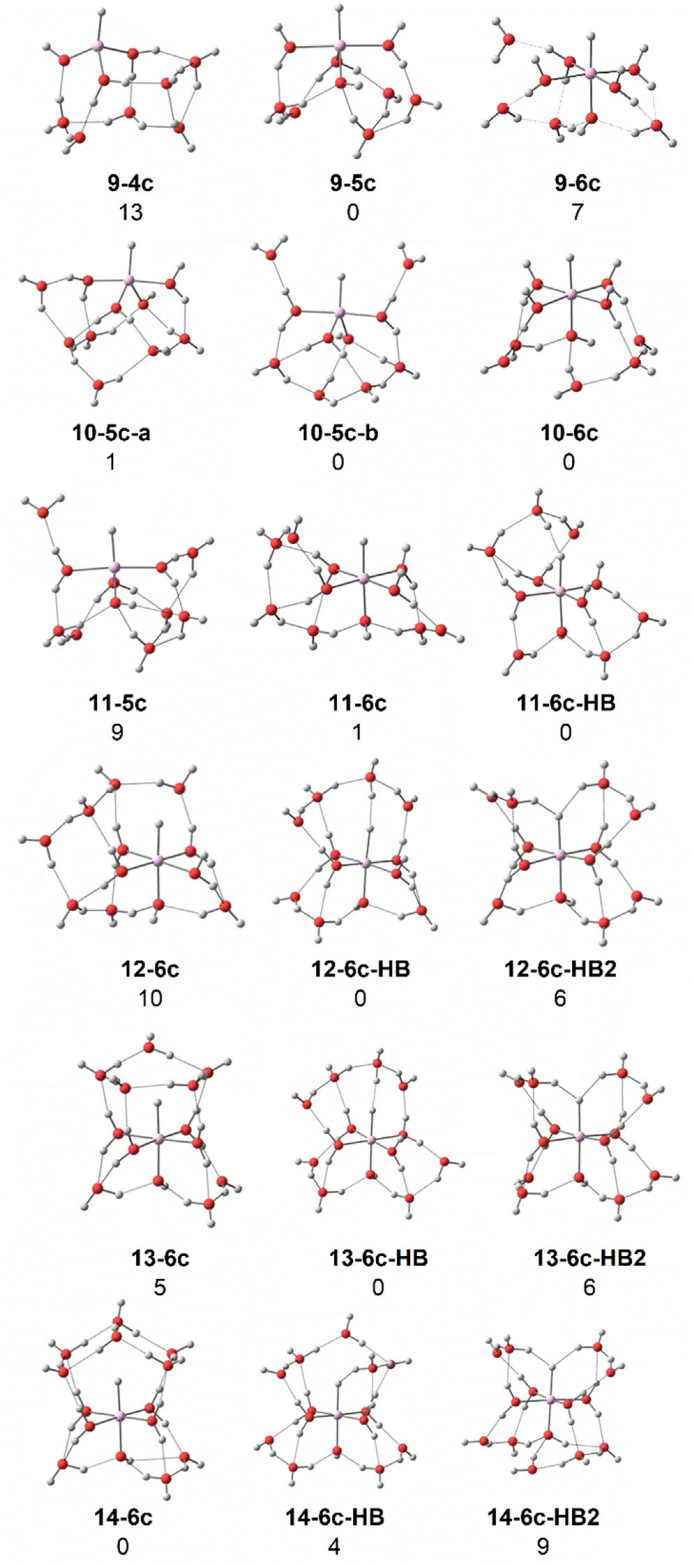
Ausgewählte energetisch niedrig liegende Strukturen von HAlOH^+^(H_2_O)_*n*−1_ für *n=*9–14. Relative Energien bei 0 K in kJ mol^−1^ wurden auf dem Theorieniveau M06/6‐311++G** berechnet.

In Abbildung 3 vergleichen wir die simulierten Spektren dieser tiefliegenden Strukturen mit den experimentellen IRMPD‐Spektren. Für *n=*9 weist der fünffach koordinierte Komplex **9‐5 c** eine freie Al‐H‐Streckschwingung auf, die mit der experimentellen Bandenposition gut übereinstimmt. Es ist auch die energetisch niedrigstliegende Struktur, die wir gefunden haben, und die Position der Hauptabsorption der Wasserbiegeschwingung wird gut reproduziert. Allerdings zeigen die berechneten Spektren eine ausgeprägte Struktur im Wasserbiegebereich, während das Experiment eine erhebliche Verbreiterung zeigt. Wir führen dies auf das Vorhandensein einer Vielzahl von Isomeren zurück, was typisch für wasserstoffgebundene Netzwerke ist.^[24]^ Die höher liegenden sechs‐ und vierfach koordinierten Komplexe, **9‐6 c** und **9‐4 c**, die eine ausgeprägte Rot‐ bzw. Blauverschiebung der Al‐H‐Streckschwingung aufweisen, werden nicht beobachtet. Bei *n=*10 werden drei isoenergetische Strukturen **10‐5 c**‐**a**, **10‐5 c**‐**b** und **10‐6 c** betrachtet. Interessanterweise verschiebt sich in der sechsfach koordinierten Struktur die Al‐H‐Streckschwingung signifikant in den roten Bereich, was die neue starke Bande bei etwa 1870 cm^−1^ erklärt. Wie von der Theorie vorhergesagt, existieren im Experiment fünf‐ und sechsfach koordinierte Strukturen mit *n=*10 nebeneinander. Mit einem weiteren Wassermolekül ist die fünffach koordinierte Infrarotsignatur für *n=*11 fast verschwunden, und der Al‐H‐Streckbereich wird, der breiten Bande nach zu urteilen, von mehreren sechsfach koordinierten Strukturisomeren mit unterschiedlichen Wellenzahlen dominiert, was auch durch DFT‐Rechnungen unterstützt wird (Abbildung S1). Gleichzeitig wird die Absorption auf der blauen Seite des Wasserbiegebereichs intensiver. Für *n=*12 dominieren die sechsfach koordinierten Strukturen deutlich, und die Al‐H‐Streckschwingung verschiebt sich leicht ins Rote und scheint an Intensität zu verlieren. Wir haben auch den O‐H‐Streckbereich untersucht, um nach der spektralen Signatur der Hydroxid‐O‐H‐Streckung zu suchen, aber es stellt sich heraus, dass diese zu der breiten Bande der wasserstoffbrückengebundenen O‐H‐Streckschwingungen beiträgt, siehe Abbildung S3 für das Spektrum von *n=*13.

**Figure 3:**
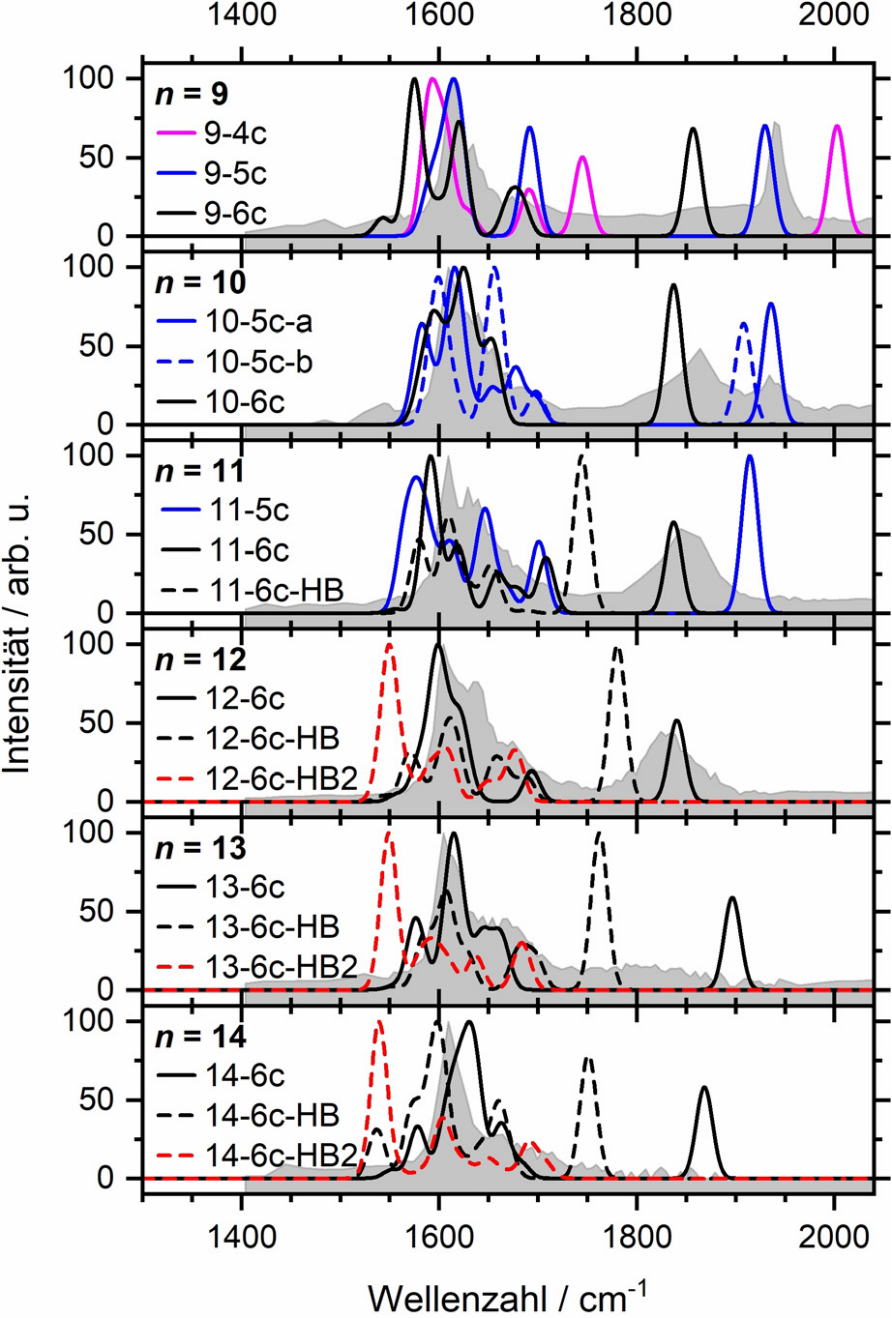
Vergleich zwischen experimentellen IRMPD‐ (grau schattierte Fläche) und theoretischen IR‐Spektren (Linien), die auf dem Theorieniveau B3LYP/6‐311++G** berechnet wurden.

Es sei erwähnt, dass bei der experimentellen Temperatur von 80 K der Beginn des Schmelzübergangs für solch kleine Cluster zu erwarten ist,^[25]^ das heißt, dass mindestens eine Wasserstoffbrückenbindung gebrochen wird, was etwa 20 kJ mol^−1^ an latenter Wärme liefert. Zusammen mit thermisch angeregten Schwingungen und internen sowie externen Rotationen liefert ein Laser‐IR‐Photon von 18–25 kJ mol^−1^ die fehlende Energie für die Verdampfung eines Wassermoleküls, die mit 51–54 kJ mol^−1^ berechnet wurde, siehe Tabelle S4. Dies stimmt mit unserer gemessenen IRMPD‐Kinetik überein, Abbildung S4, die auf einen Ein‐Photonen‐Prozess hinweist.

Interessanterweise werden Strukturen, die eine Wasserstoffbrückenbindung zum Hydrid aufweisen, bei *n=*11 energetisch konkurrenzfähig, mit einer starken Rotverschiebung der Al‐H‐Streckschwingung. Die genaue Position der Al‐H‐Streckschwingung ist jedoch sehr empfindlich gegenüber der Struktur des Wasserstoffbrückenbindungsnetzwerks (Schema 1). Außerdem zeigen Doppelakzeptorstrukturen wie **12‐6 c‐HB2** eine extreme Rotverschiebung. Für *n=*13 können Strukturen, bei denen das Hydrid als Einzelakzeptor wirkt, die breite Absorption bei ca. 1720–1900 cm^−1^ erklären, während die Banden von Doppelakzeptorstrukturen unterhalb von 1580 cm^−1^ verschmiert sind. Mit jedem zusätzlichen Wassermolekül steigt die Anzahl der energetisch zugänglichen Clusterisomere. Gleichzeitig deckt die Position der Al‐H‐Bande in den ***n*** **‐6 c‐HB2**‐Strukturen einen Spektralbereich von 200 cm^−1^ ab. Diese beiden Effekte zusammen erklären, warum kein einzelner starker Peak oder eine gleichmäßige Bande im Spektrum erscheint, die den ***n*** **‐6 c‐HB2**‐Strukturen zugeordnet werden könnte.

**Scheme 1:**
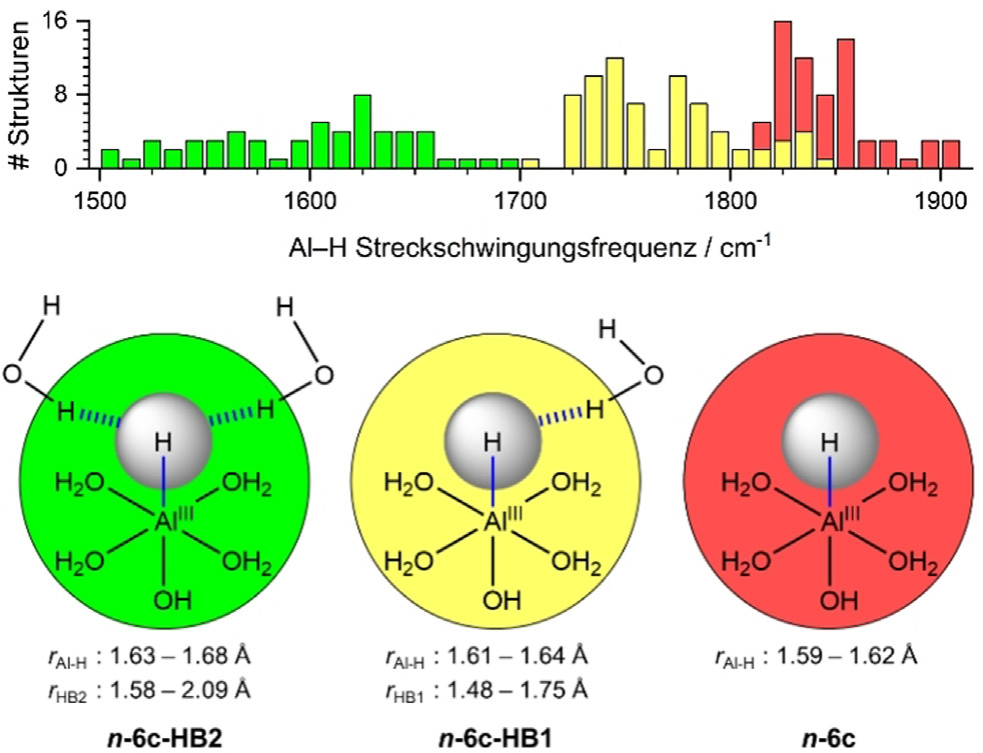
Zusammenfassung der Frequenzen der Al‐H‐Streckschwingung, die auf dem B3LYP/6‐311++G**‐Niveau berechnet wurden. Der Al‐H‐Abstand *r*_Al‐H_ und die Wasser‐Hydrid‐Wasserstoffbrückenbindungsabstände *r*_HB1_ und *r*_HB2_ wurden auf dem M06/6‐311++G**‐Niveau optimiert, einschließlich aller Geometrien von sechsfach koordiniertem ***n*** **‐6 c**, ***n*** **‐6 c‐HB** und ***n*** **‐6 c‐HB2** für *n=*11–14. Die Geometrieparameter und die Frequenzen der Al‐H‐Streckschwingung aller berechneten Strukturen sind in den Hintergrundinformationen, Tabellen S1–S3, verfügbar. Das Histogramm (oben) zeigt, wie viele Strukturen in jedem Frequenzbereich gefunden wurden.

Normalerweise wird eine Wasserstoffbrücke zwischen einem Wasserstoffatom des Donors und einem freien Elektronenpaar des Akzeptors gebildet, das ein sp^3^‐hybridisiertes Orbital besetzt. Im vorliegenden Fall fungiert ein Elektronenpaar am sphärisch symmetrischen s‐Orbital des Hydrids als Akzeptor für zwei Wasserstoffbrückenbindungen, wie in der Abbildung 4 dargestellt wird. Diese symmetrische Aufteilung des Akzeptor‐Elektronenpaares schwächt beide Wasserstoffbrückenbindungen, ersichtlich an deren deutlich vergrößerten Bindungslängen in den ***n*** **‐6 c‐HB2**‐Isomeren, dargestellt in Schema 1. Die kooperativen Effekte der Wasserstoffbrückenbindungen verlängern progressiv den Al‐H‐Abstand ***n*** **‐6 c**< ***n*** **‐6 c‐HB**< ***n*** **‐6 c‐HB2**, was die Bindungsschwächung anzeigt, die sich in der deutlichen Rotverschiebung widerspiegelt.

**Figure 4:**
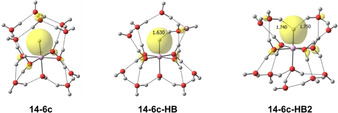
Höchste besetzte Molekülorbitale (HOMOs) von **14‐6 c**, **14‐6 c‐HB** und **14‐6 c‐HB2** mit einem Isowert von 0,05 a.u., erhalten auf dem M06/6‐311++G**‐Niveau. Das HOMO repräsentiert das 1s^2^‐Elektronenpaar am Hydrid, das als Einfach‐ oder Doppel‐Wasserstoffbrücken‐Akzeptor fungiert.

Es ist bemerkenswert, dass das Auftreten der wasserstoffgebundenen Al‐H, was im Experiment durch das Verschwinden der freien Al‐H‐Strecke deutlich wird, mit dem Beginn der H_2_‐Bildung zusammenfällt. Während Strukturen, die eine Hydrid‐Wasserstoffbindung enthalten, für die kleineren Cluster (*n=*9, 10) optimiert werden können, sind die jeweiligen Minima sehr flach, und die Cluster relaxieren zu nicht‐wasserstoffbrückengebundenen Strukturen, wenn die Geometrie leicht verzerrt ist. Dies deutet darauf hin, dass bei der experimentellen Temperatur von 80 K diese Strukturen nicht signifikant vorkommen, was erklärt, warum die H_2_‐Bildung für diese Größen nicht beobachtet wird. Die Integration des Hydrids in das wasserstoffgebundene Netzwerk des Clusters ist eine Voraussetzung für die Wasserstoffentwicklung. Unsere Ergebnisse unterstützen den theoretisch vorhergesagten Mechanismus der H_2_‐Bildung stark. Außerdem zeigen wir, dass ein Metallhydrid ein sehr guter Akzeptor für Wasserstoffbrückenbindungen ist. Sauerstoff in Wasser mit seinen zwei freien Elektronenpaaren kann als Doppelakzeptor wirken, während Stickstoff, z. B. in Ammoniak, mit nur einem freien Elektronenpaar immer ein Einzelakzeptor ist. Hydrid, mit zwei Elektronen in einem sphärisch symmetrischen s‐Typ‐Molekülorbital, kann sogar als Doppelakzeptor in einem Wasserstoffbrückenbindungsnetzwerk wirken.

## Conflict of interest

Die Autoren erklären, dass keine Interessenkonflikte vorliegen.

## Supporting information

As a service to our authors and readers, this journal provides supporting information supplied by the authors. Such materials are peer reviewed and may be re‐organized for online delivery, but are not copy‐edited or typeset. Technical support issues arising from supporting information (other than missing files) should be addressed to the authors.

Supplementary

## References

[1a] M. Carmo , D. L. Fritz , J. Mergel , D. Stolten , Int. J. Hydrogen Energy 2013, 38, 4901;

[1b] M. Gong , D.-Y. Wang , C.-C. Chen , B.-J. Hwang , H. Dai , Nano Res. 2016, 9, 28.

[2] C. Jiang , S. J. A. Moniz , A. Wang , T. Zhang , J. Tang , Chem. Soc. Rev. 2017, 46, 4645. 28644493 10.1039/c6cs00306k

[3] H. Zhang , H. Wu , Y. Jia , B. Yin , L. Geng , Z. Luo , K. Hansen , Commun. Chem. 2020, 3, 148. 36703429 10.1038/s42004-020-00396-9 PMC9814650

[4] C. J. Mundy , J. Hutter , M. Parrinello , J. Am. Chem. Soc. 2000, 122, 4837.

[5a] R. C. Dunbar , Mass Spectrom. Rev. 2004, 23, 127; 14732935 10.1002/mas.10074

[5b] T. Schindler , C. Berg , G. Niedner-Schatteburg , V. E. Bondybey , Chem. Phys. Lett. 1996, 250, 301;

[5c] D. Thoelmann , D. S. Tonner , T. B. McMahon , J. Phys. Chem. 1994, 98, 2002;

[5d] M. Sena , J. M. Riveros , Rapid Commun. Mass Spectrom. 1994, 8, 1031;

[5e] B. S. Fox , M. K. Beyer , V. E. Bondybey , J. Phys. Chem. A 2001, 105, 6386.

[6a] C. Berg , U. Achatz , M. Beyer , S. Joos , G. Albert , T. Schindler , G. Niedner-Schatteburg , V. E. Bondybey , Int. J. Mass Spectrom. Ion Process. 1997, 167/168, 723;

[6b] C. Berg , M. Beyer , U. Achatz , S. Joos , G. Niedner-Schatteburg , V. E. Bondybey , Chem. Phys. 1998, 239, 379.

[7] M. Beyer , C. Berg , H. W. Görlitzer , T. Schindler , U. Achatz , G. Albert , G. Niedner-Schatteburg , V. E. Bondybey , J. Am. Chem. Soc. 1996, 118, 7386.

[8] M. Beyer , U. Achatz , C. Berg , S. Joos , G. Niedner-Schatteburg , V. E. Bondybey , J. Phys. Chem. A 1999, 103, 671.

[9] B. S. Fox , I. Balteanu , O. P. Balaj , H. C. Liu , M. K. Beyer , V. E. Bondybey , Phys. Chem. Chem. Phys. 2002, 4, 2224.

[10a] F. Misaizu , M. Sanekata , K. Tsukamoto , K. Fuke , S. Iwata , J. Phys. Chem. 1992, 96, 8259;

[10b] K. Fuke , K. Hashimoto , S. Iwata , Adv. Chem. Phys. 1999, 110, 431;

[10c] F. Misaizu , M. Sanekata , K. Fuke , S. Iwata , J. Chem. Phys. 1994, 100, 1161;

[10d] M. Ončák , T. Taxer , E. Barwa , C. van der Linde , M. K. Beyer , J. Chem. Phys. 2018, 149, 044309. 30068190 10.1063/1.5037401 PMC7075709

[11] B. Scharfschwerdt , C. van der Linde , O. P. Balaj , I. Herber , D. Schütze , M. K. Beyer , Low Temp. Phys. 2012, 38, 717.

[12] B. M. Reinhard , G. Niedner-Schatteburg , J. Phys. Chem. A 2002, 106, 7988.

[13] C.-K. Siu , Z.-F. Liu , J. S. Tse , J. Am. Chem. Soc. 2002, 124, 10846. 12207540 10.1021/ja0117579

[14] H. Watanabe , S. Iwata , J. Phys. Chem. 1996, 100, 3377.

[15a] Z. Sun , C.-K. Siu , O. P. Balaj , M. Gruber , V. E. Bondybey , M. K. Beyer , Angew. Chem. Int. Ed. 2006, 45, 4027; 10.1002/anie.200504135 16683283

[15b] C. van der Linde , M. K. Beyer , Phys. Chem. Chem. Phys. 2011, 13, 6776; 21399775 10.1039/c1cp00048a

[15c] C. van der Linde , M. K. Beyer , J. Phys. Chem. A 2012, 116, 10676; 23075152 10.1021/jp308744p

[15d] P. U. Andersson , M. J. Ryding , O. Sekiguchi , E. Uggerud , Phys. Chem. Chem. Phys. 2008, 10, 6127; 18846302 10.1039/b804584d

[15e] M. Ryding , P. Andersson , A. Zatula , E. Uggerud , Eur. J. Mass Spectrom. 2012, 18, 215. 10.1255/ejms.1172 22641727

[16a] J. Oomens , B. G. Sartakov , G. Meijer , G. von Helden , Int. J. Mass Spectrom. 2006, 254, 1;

[16b] L. Jašíková , J. Roithová , Chem. Eur. J. 2018, 24, 3374; 29314303 10.1002/chem.201705692

[16c] H. Schwarz , K. R. Asmis , Chem. Eur. J. 2019, 25, 2112; 30623993 10.1002/chem.201805836

[16d] L. MacAleese , P. Maître , Mass Spectrom. Rev. 2007, 26, 583; 17471578 10.1002/mas.20138

[16e] N. Heine , K. R. Asmis , Int. Rev. Phys. Chem. 2015, 34, 1;

[16f] M. A. Duncan , Int. Rev. Phys. Chem. 2003, 22, 407.

[17a] C. Berg , T. Schindler , G. Niedner-Schatteburg , V. E. Bondybey , J. Chem. Phys. 1995, 102, 4870;

[17b] M. A. Duncan , Rev. Sci. Instrum. 2012, 83, 041101. 22559508 10.1063/1.3697599

[18] O. P. Balaj , C. B. Berg , S. J. Reitmeier , V. E. Bondybey , M. K. Beyer , Int. J. Mass Spectrom. 2009, 279, 5.

[19] X. Wang , L. Andrews , S. Tam , M. E. DeRose , M. E. Fajardo , J. Am. Chem. Soc. 2003, 125, 9218. 15369378 10.1021/ja0353560

[20a] M. J. Frisch, G. W. Trucks, H. B. Schlegel, G. E. Scuseria, M. A. Robb, J. R. Cheeseman, G. Scalmani, V. Barone, G. A. Petersson, H. Nakatsuji et al., *Gaussian 16 Revision B.01*, **2016**;

[20b] A. D. Becke , J. Chem. Phys. 1993, 98, 5648;

[20c] C. T. Lee , W. T. Yang , R. G. Parr , Phys. Rev. B 1988, 37, 785. 10.1103/physrevb.37.785 9944570

[21] W. K. Tang , X. Mu , M. Li , J. Martens , G. Berden , J. Oomens , I. K. Chu , C.-K. Siu , Phys. Chem. Chem. Phys. 2020, 22, 21393. 32940309 10.1039/d0cp00533a

[22] C. L. Moss , J. Chamot-Rooke , E. Nicol , J. Brown , I. Campuzano , K. Richardson , J. P. Williams , M. F. Bush , B. Bythell , B. Paizs , et al., J. Phys. Chem. B 2012, 116, 3445. 22364440 10.1021/jp3000784

[23] Y. Zhao , D. G. Truhlar , Theor. Chem. Acc. 2008, 120, 215.

[24] N. Yang , C. H. Duong , P. J. Kelleher , M. A. Johnson , Nat. Chem. 2020, 12, 159. 31767995 10.1038/s41557-019-0376-9

[25a] C. Hock , M. Schmidt , R. Kuhnen , C. Bartels , L. Ma , H. Haberland , B. von Issendorff , Phys. Rev. Lett. 2009, 103, 73401; 10.1103/PhysRevLett.103.073401 19792643

[25b] M. Schmidt , B. von Issendorff , J. Chem. Phys. 2012, 136, 164307. 22559482 10.1063/1.4705266

