# Supplementary material for: Auf zur Wasserstoffentwicklung: Das Infrarot‐Spektrum von hydratisiertem Aluminiumhydrid‐Hydroxid HAlOH+(H2O) n−1, n=9–14
Source: Angew Chem Weinheim Bergstr Ger. 2021 Jun 4;133(31):16994–9. doi: 10.1002/ange.202105166 (PMC10947318; doi:10.1002/ange.202105166)
Supplement: Supplementary file 1 — Supplementary [file ANGE-133-16994-s001.pdf]

Angewandte Chemie
Eine Zeitschrift der Gesellschaft Deutscher Chemiker

Supporting Information

Getting Ready for the Hydrogen Evolution Reaction: The Infrared Spectrum of Hydrated Aluminum Hydride–Hydroxide $HAlOH^+(H_2O)_{n-1}$, $n = 9$–14

Jakob Heller+, Wai Kit Tang+, Ethan M. Cunningham, Ephrem G. Demissie, Christian van der Linde, Wing Ka Lam, Milan Ončák, Chi-Kit Siu, and Martin K. Beyer**

ange_202105166_sm_miscellaneous_information.pdf

Contents

Experimental Details

Computational Details

Figures S1-S4

Tables S1-S4

Computational Results on Optimized Geometries Discussed in the Main Text

Experimental Details

The experiments are performed on a Bruker Spectrospin 4.7 Tesla Fourier-Transformation Ion Cyclotron Resonance Mass Spectrometer (FT-ICR MS).[1] The mass spectrometer is equipped with a Bruker infinity cell[2] along with a laser vaporization source,[3] where a solid disk of aluminum is vaporized by a frequency doubled Litron Nano S 60-30 Nd:YAG laser (532 nm, 5 mJ/pulse, 30 Hz). The plasma containing Al^+ is entrained in a pulse of Helium seeded with water vapor created *via* a homebuilt piezoelectric valve. The ensuing pulse is cooled *via* supersonic expansion, whereby hydrated aluminum complexes, $Al^+(H_2O)_n$, are created. The clusters are transferred into the ICR-Cell, where they are mass selected and stored within the 4.7 T magnetic field[4] under ultra-high vacuum conditions ($p \approx 5 \times 10^{-10}$ mbar).

The mass-selected ion of interest is irradiated with infrared light provided by an EKPLSA NT273-XIR OPO covering the range from 1400–2240 cm^{-1}. Typical irradiation times are 0.2 s at 1000 Hz repetition rate. The normalized IRMPD Yield is calculated from the precursor ion and fragment ion intensities along with laser power are considered. The laser power is measured after every mass spectrum to account for any fluctuations. Especially for larger clusters, dissociation due to black-body infrared radiative dissociation (BIRD) from the cell walls can occur. To prevent this, the ICR cell is surrounded by a copper jacket, whereby the ions can be cooled with liquid nitrogen to a temperature of *ca.* 85 K,[5] minimizing the effects due to BIRD.[6,7] The remaining BIRD fragmentation can be compensated by subtraction of the measured fragment ion intensities with a reference mass spectrum where the ions were trapped without irradiation from the OPO. In concordance with previous investigations, we expect these clusters to be thermalized within the ICR cell, since IR exchange in the 300–500 cm^{-1} range is efficient, and there are many IR photons present at 85 K.[7]

Computational Details

All geometries of $HAlOH^+(H_2O)_{n-1}$ were optimized at M06/6-311++G** level of theory and confirmed as local minima with no imaginary frequency evaluated at the same level of theory. Initially, the penta- and hexa-coordinate ionic cores (i.e., $HAlOH^+(H_2O)_3$ and $HAlOH^+(H_2O)_4$) were optimized. From these smallest cores (n = 4 and 5, respectively), a water molecule was then placed at different locations in the second solvation shell in a way that maximizes the number of hydrogen bonds among water molecules, followed by further geometry optimizations. This procedure was repeated systematically by placing additional water molecules to low-lying geometries of the n-1 cluster, to build plausible initial geometries for clusters up to n = 14 for both coordinate modes. All optimized geometries at each size $9 \leq n \leq 14$ were classified into three categories: conformers with the hydride of the Al-H bond forming (i) zero (***n*-5c** and ***n*-6c**), (ii) one (***n*-5c-HB** and ***n*-6c-HB**) or (iii) two (***n*-5c-HB2** and ***n*-6c-HB2**) hydrogen bonds. To obtain vibrational spectra of these conformers, their geometries were re-optimized, followed by harmonic frequency analyses at the B3LYP/6-311++G** level of theory. The Al-H bond lengths (r_{AlH}) and hydrogen bond lengths between the hydride and water molecules (r_{HB1} and r_{HB2}) (obtained from M06) along with the Al-H stretching frequency (obtained from B3LYP with an anharmonic scaling of 0.982) are listed in Tables S1–S3.

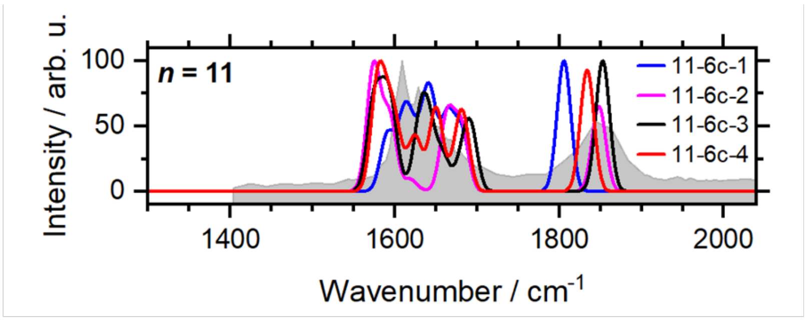

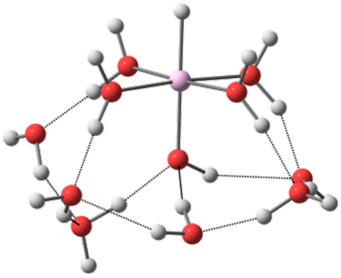

11-6c-1
6

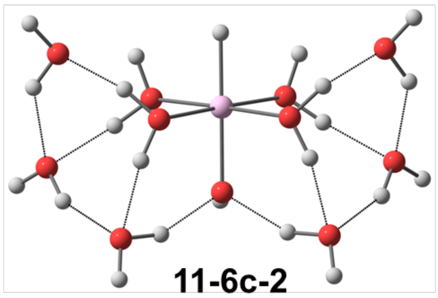

11-6c-2
5

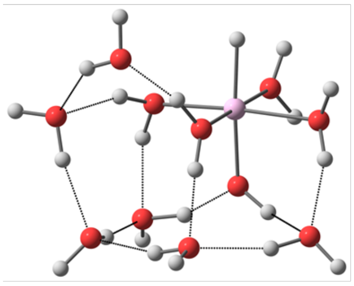

11-6c-3
15

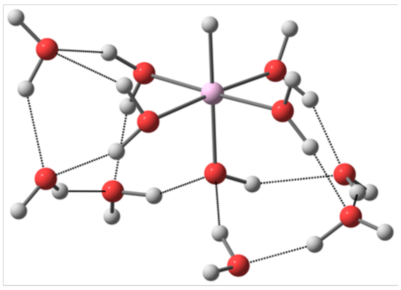

11-6c-4
7

Figure S1. Selected six-coordinate conformers of $HAlOH^+(H_2O)_{n-1}$ (n = 11) optimized at the M06/6-311++G** level. Relative zero-point corrected energies ΔE_0 are in kJ mol^{-1}, referenced to the structures in Figure 2. Theoretical IR spectra (colored lines) were obtained from harmonic vibration analyses including an anharmonic scaling factor of 0.982 for geometries optimized at the B3LYP/6-311++G** level. Calculated lines are broadened with Gaussian functions with 20 cm^{-1} FWHM. The experimental IRMPD spectrum is shown by the gray shaded area.

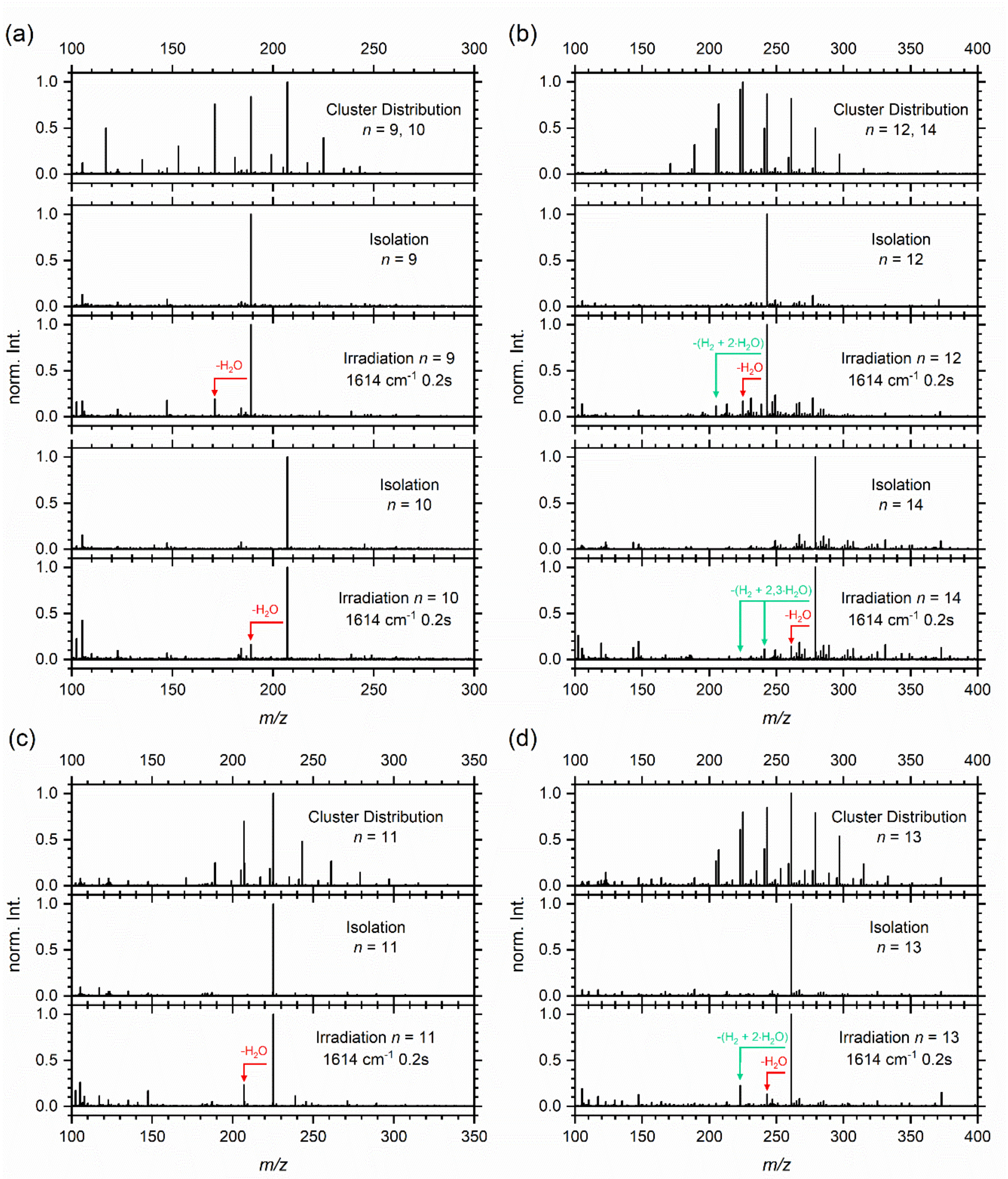

Figure S2. Mass spectra of a) *n* = 9 and 10, b) *n* = 12 and 14, c) *n* = 11, and d) *n* = 13. Taking a) as a representative, the top panel represents the cluster distribution from the ion source, followed by the mass-selected ion of interest, *n* = 9. Mass-selection was achieved in the ICR cell *via* resonant excitation and ejection of all other unwanted ions.[4] To avoid unnecessarily long delays prior to IR irradiation and to minimize potential residual excitation of the ion of interest, we did not employ broad-band excitation, and we did not eject some low-intensity peaks that do not interfere with the experiment. Each mass-selected ion was irradiated for 0.2 s at 1614 cm^{-1}. For *n* = 9, 10 and 11, a) and c), IRMPD leads to loss of intact water molecules, whereas for *n* = 12, 14 and 13, b) and d), loss of H_2+xH_2O, x = 2,3, is also observed.

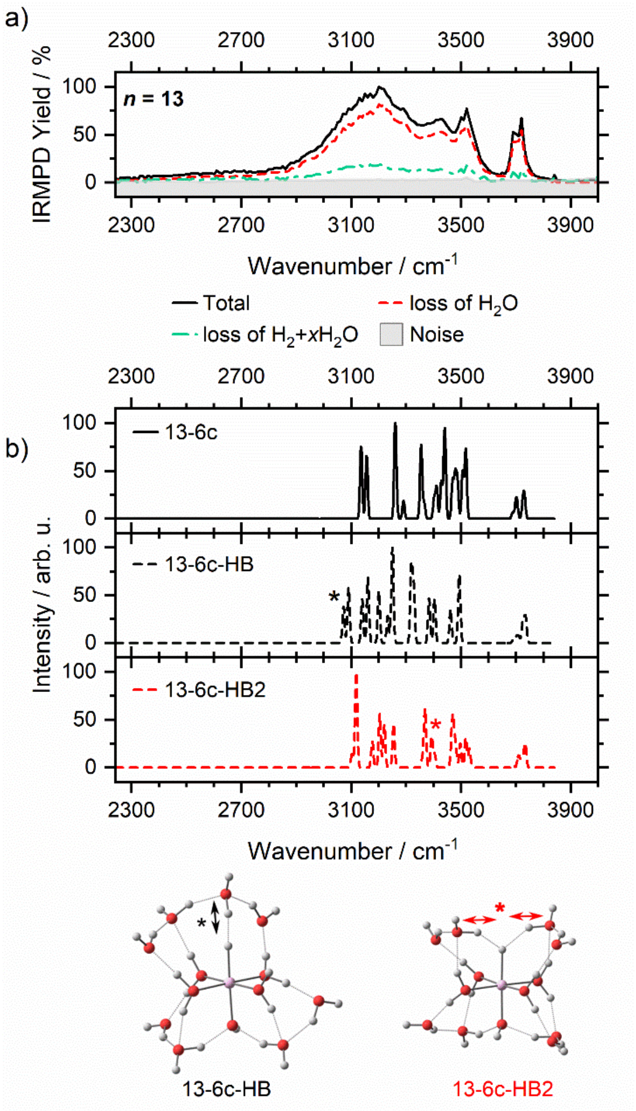

Figure S3. a) Experimental IRMPD spectrum of $n = 13$ in the O-H stretch region. b) Simulated IR spectra obtained from harmonic vibration analyses including an anharmonic scaling factor of 0.96 for geometries optimized at the B3LYP/6-311++G** level. The hydrogen-bonded hydride O-H stretch is indicated by an asterisk (*).

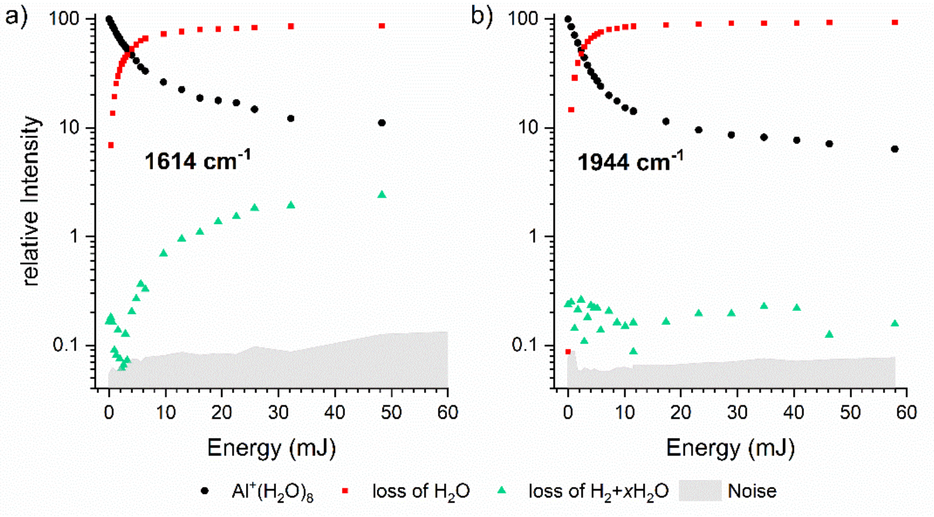

Figure S4: IRMPD kinetics of $HAlOH^+(H_2O)_7$ a) 1614 cm^{-1} and b) 1944 cm^{-1} scaled to the total energy provided to the ion cloud to compensate for the wavelength-dependent laser power. The linear onset of the decay of the reactant ion in the semilogarithmic plot, without exhibiting an induction delay, indicates a single-photon process. The upward curvature at later times is assigned to incomplete overlap of the laser beam with the ion cloud. Absorption of multiple photons would lead to a downward curvature.

Table S1. Summary of Al-H bond lengths r_{AlH}, relative zero-point corrected energies ΔE_0 referenced to Figure 2 (calculated at the M06/6-311++G** level of theory) and Al-H stretching frequencies (calculated at the B3LYP/6-311++G** level of theory with an anharmonic scaling of 0.982) for all optimized hexa-coordinate geometries featuring a free Al-H bond without forming hydrogen bonds (***n*-6c**).

Structure Label	Al-H length r_{AlH} / Å	ΔE_0 / kJ mol^{-1}	Al-H stretch freq. / cm^{-1}
**9-6c**	1.60	7	1858
**9-6c-1**	1.59	34	1843
**9-6c-2**	1.59	10	1851
**9-6c-3**	1.60	17	1840
**9-6c-4**	1.59	22	1863
**10-6c**	1.60	0	1837
**10-6c-1**	1.59	12	1855
**10-6c-2**	1.60	6	1853
**10-6c-3**	1.59	5	1849
**10-6c-4**	1.59	11	1849
**10-6c-5**	1.60	8	1830
**10-6c-6**	1.59	8	1859
**10-6c-7**	1.59	4	1851
**10-6c-8**	1.59	9	1850
**10-6c-9**	1.60	16	1827
**10-6c-10**	1.60	24	1829
**11-6c**	1.60	1	1837
**11-6c-1**	1.60	16	1803
**11-6c-2**	1.60	6	1805
**11-6c-3**	1.60	25	1869
**11-6c-4**	1.60	19	1835
**11-6c-5**	1.60	12	1830
**11-6c-6**	1.60	10	1832
**11-6c-7**	1.59	18	1852
**11-6c-8**	1.60	7	1833
**11-6c-9**	1.60	5	1848
**11-6c-10**	1.60	14	1844
**11-6c-11**	1.60	16	1825
**11-6c-12**	1.60	13	1829
**11-6c-13**	1.59	13	1897
**11-6c-14**	1.60	23	1835
**11-6c-15**	1.60	25	1824
**11-6c-16**	1.60	28	1820
**12-6c**	1.60	10	1840
**12-6c-1**	1.60	13	1825
**12-6c-2**	1.60	13	1825
**12-6c-3**	1.60	15	1818
**12-6c-4**	1.59	10	1876
**12-6c-5**	1.60	13	1825
**12-6c-6**	1.60	15	1839
**12-6c-7**	1.60	13	1825
**12-6c-8**	1.60	16	1828
**12-6c-9**	1.59	13	1873
**12-6c-10**	1.59	18	1902
**12-6c-11**	1.59	21	1863
**12-6c-12**	1.60	22	1825
**12-6c-13**	1.59	17	1859
**12-6c-14**	1.60	13	1825
**13-6c**	1.59	5	1896
**13-6c-1**	1.60	20	1833
**13-6c-2**	1.60	19	1822
**13-6c-3**	1.60	16	1816
**13-6c-4**	1.60	15	1822
**13-6c-5**	1.60	15	1877
**13-6c-6**	1.61	18	1857
**13-6c-7**	1.60	25	1830
**13-6c-8**	1.60	34	1836
**13-6c-9**	1.59	28	1903
**13-6c-10**	1.61	20	1850
**13-6c-11**	1.60	29	1856
**13-6c-12**	1.61	21	1812
**13-6c-13**	1.61	20	1832
**14-6c**	1.61	0	1868
**14-6c-1**	1.60	21	1816
**14-6c-2**	1.60	18	1816
**14-6c-3**	1.60	5	1892
**14-6c-4**	1.60	9	1892
**14-6c-5**	1.61	7	1854
**14-6c-6**	1.61	23	1880
**14-6c-7**	1.60	20	1903
**14-6c-8**	1.61	14	1844
**14-6c-9**	1.62	7	1823
**14-6c-10**	1.61	33	1856
**14-6c-11**	1.61	8	1835
**14-6c-12**	1.61	29	1844
**Range**	1.59 – 1.62	0 – 34	1803 – 1903

Table S2. Summary of Al-H bond lengths r_{AlH}, hydrogen-bond lengths between the hydride of Al–H and water molecules r_{HB1}, relative zero-point corrected energies ΔE_0 referenced to Figure 2 (calculated at the M06/6-311++G** level of theory) and Al-H stretching frequencies (calculated at the B3LYP/6-311++G** level of theory with an anharmonic scaling of 0.982) for all optimized hexa-coordinate geometries featuring a single hydrogen bond to Al-H (***n*-6c-HB1**).

Structure Label	Al-H length r_{AlH} / Å	H-bond length r_{HB1} / Å	ΔE_0 / kJ mol^{-1}	Al-H stretch freq. / cm^{-1}
**11-6c-HB**	1.63	1.52	0	1744
**11-6c-HB-1**	1.62	1.59	6	1741
**11-6c-HB-2**	1.62	1.60	5	1747
**11-6c-HB-3**	1.62	1.56	7	1785
**11-6c-HB-4**	1.61	1.66	23	1786
**11-6c-HB-5**	1.63	1.52	0	1754
**11-6c-HB-6**	1.62	1.66	22	1785
**11-6c-HB-7**	1.62	1.56	17	1807
**11-6c-HB-8**	1.62	1.53	25	1779
**11-6c-HB-9**	1.62	1.71	5	1735
**11-6c-HB-10**	1.62	1.56	17	1826
**11-6c-HB-11**	1.62	1.60	9	1794
**11-6c-HB-12**	1.62	1.55	29	1833
**11-6c-HB-13**	1.64	1.48	31	1757
**11-6c-HB-14**	1.62	1.61	17	1735
**11-6c-HB-15**	1.61	1.58	16	1812
**11-6c-HB-16**	1.62	1.52	36	1836
**11-6c-HB-17**	1.63	1.52	14	1781
**11-6c-HB-18**	1.62	1.75	6	1722
**11-6c-HB-19**	1.63	1.69	17	1709
**12-6c-HB**	1.63	1.54	0	1780
**12-6c-HB-1**	1.62	1.72	9	1733
**12-6c-HB-2**	1.63	1.58	9	1722
**12-6c-HB-3**	1.62	1.67	1	1736
**12-6c-HB-4**	1.63	1.64	13	1773
**12-6c-HB-5**	1.63	1.55	2	1783
**12-6c-HB-6**	1.63	1.59	27	1722
**12-6c-HB-7**	1.63	1.58	9	1723
**12-6c-HB-8**	1.61	1.55	26	1823
**12-6c-HB-9**	1.62	1.63	9	1773
**12-6c-HB-10**	1.61	1.54	38	1846
**12-6c-HB-11**	1.62	1.69	36	1752
**12-6c-HB-12**	1.63	1.61	12	1738
**12-6c-HB-13**	1.63	1.64	32	1743
**12-6c-HB-14**	1.62	1.56	36	1821
**12-6c-HB-15**	1.63	1.64	26	1743
**12-6c-HB-16**	1.63	1.56	20	1781
**12-6c-HB-17**	1.62	1.57	26	1811
**12-6c-HB-18**	1.63	1.64	18	1738
**13-6c-HB**	1.64	1.54	0	1762
**13-6c-HB-1**	1.62	1.58	19	1747
**13-6c-HB-2**	1.63	1.59	5	1732
**13-6c-HB-3**	1.63	1.56	11	1755
**13-6c-HB-4**	1.62	1.61	6	1775
**13-6c-HB-5**	1.63	1.54	2	1768
**13-6c-HB-6**	1.63	1.59	12	1764
**13-6c-HB-7**	1.62	1.57	21	1839
**13-6c-HB-8**	1.63	1.62	21	1754
**13-6c-HB-9**	1.63	1.60	31	1741
**13-6c-HB-10**	1.63	1.54	22	1792
**13-6c-HB-11**	1.62	1.74	28	1794
**13-6c-HB-12**	1.62	1.64	15	1775
**13-6c-HB-13**	1.62	1.67	23	1720
**13-6c-HB-14**	1.63	1.55	21	1746
**13-6c-HB-15**	1.63	1.59	24	1794
**13-6c-HB-16**	1.62	1.53	19	1804
**13-6c-HB-17**	1.62	1.57	26	1839
**13-6c-HB-18**	1.63	1.57	22	1757
**14-6c-HB**	1.63	1.63	4	1751
**14-6c-HB-1**	1.63	1.58	16	1745
**14-6c-HB-2**	1.63	1.61	30	1754
**14-6c-HB-3**	1.63	1.58	25	1730
**14-6c-HB-4**	1.63	1.58	27	1721
**14-6c-HB-5**	1.63	1.64	30	1725
**14-6c-HB-6**	1.63	1.58	27	1779
**14-6c-HB-7**	1.62	1.58	16	1783
**14-6c-HB-8**	1.63	1.53	19	1773
**14-6c-HB-9**	1.63	1.65	18	1723
**14-6c-HB-10**	1.63	1.63	4	1736
**14-6c-HB-11**	1.62	1.73	25	1736
**14-6c-HB-12**	1.63	1.54	5	1776
**14-6c-HB-13**	1.63	1.57	15	1745
**14-6c-HB-14**	1.64	1.53	13	1742
**14-6c-HB-15**	1.64	1.50	8	1742
**14-6c-HB-16**	1.63	1.60	22	1777
**14-6c-HB-17**	1.64	1.52	12	1742
**14-6c-HB-18**	1.62	1.60	10	1770
**Range**	1.61 – 1.64	1.48 – 1.75	0 – 38	1709 – 1846

Table S3. Summary of Al-H bond lengths r_{AlH}, hydrogen-bond lengths between the hydride of Al-H and water molecules r_{HB2}, relative zero-point corrected energies ΔE_0 referenced to Figure 2 (calculated at the M06/6-311++G** level of theory) and Al-H stretch frequencies (calculated at the B3LYP/6-311++G** level of theory with an anharmonic scaling of 0.982) for all optimized hexa-coordinate geometries featuring two hydrogen bonds to Al-H (***n*-6c-HB2**).

Structure Label	Al-H length r_{AlH} / Å	H-bond length r_{HB2} / Å		ΔE_0 / kJ mol^{-1}	Al-H stretch freq. / cm^{-1}
**11-6c-HB2-1**	1.65	1.82	1.82	32	1602
**11-6c-HB2-2**	1.65	1.79	1.79	21	1613
**11-6c-HB2-3**	1.64	1.89	1.97	12	1659
**11-6c-HB2-4**	1.63	1.78	1.96	53	1629
**11-6c-HB2-5**	1.67	1.63	1.71	47	1551
**11-6c-HB2-6**	1.65	1.92	1.94	10	1627
**11-6c-HB2-7**	1.64	1.86	1.93	21	1642
**11-6c-HB2-8**	1.64	1.92	1.98	16	1689
**11-6c-HB2-9**	1.66	1.73	1.88	45	1560
**12-6c-HB2**	1.66	1.77	1.79	6	1606
**12-6c-HB2-1**	1.64	1.87	2.00	19	1621
**12-6c-HB2-2**	1.64	1.87	1.88	12	1636
**12-6c-HB2-3**	1.65	1.75	2.08	16	1649
**12-6c-HB2-4**	1.65	1.64	2.08	16	1649
**12-6c-HB2-5**	1.66	1.67	1.73	41	1605
**12-6c-HB2-6**	1.67	1.58	1.72	27	1579
**12-6c-HB2-7**	1.67	1.64	1.72	36	1543
**12-6c-HB2-8**	1.65	1.87	1.94	11	1637
**12-6c-HB2-9**	1.64	1.84	1.96	24	1658
**12-6c-HB2-10**	1.64	1.94	1.96	9	1656
**12-6c-HB2-11**	1.65	1.75	2.09	16	1649
**12-6c-HB2-12**	1.68	1.61	1.77	16	1510
**12-6c-HB2-13**	1.66	1.74	1.81	47	1500
**13-6c-HB2**	1.66	1.74	1.76	6	1549
**13-6c-HB2-1**	1.65	1.75	2.03	13	1621
**13-6c-HB2-2**	1.65	1.72	2.08	29	1617
**13-6c-HB2-3**	1.67	1.77	1.78	28	1532
**13-6c-HB2-4**	1.66	1.63	1.87	37	1578
**13-6c-HB2-5**	1.64	1.76	1.81	31	1614
**13-6c-HB2-6**	1.67	1.63	1.64	33	1525
**13-6c-HB2-7**	1.66	1.59	1.75	41	1624
**13-6c-HB2-8**	1.64	1.86	1.88	24	1654
**13-6c-HB2-9**	1.68	1.64	1.71	33	1630
**13-6c-HB2-10**	1.65	1.85	1.87	33	1605
**13-6c-HB2-11**	1.65	1.86	1.88	20	1597
**13-6c-HB2-12**	1.65	1.89	1.89	38	1634
**13-6c-HB2-13**	1.63	1.94	2.00	23	1699
**13-6c-HB2-14**	1.65	1.74	2.04	13	1621
**13-6c-HB2-15**	1.66	1.73	1.75	32	1550
**14-6c-HB2**	1.67	1.74	1.75	9	1539
**14-6c-HB2-1**	1.65	1.65	1.85	28	1621
**14-6c-HB2-2**	1.65	1.83	1.87	31	1608
**14-6c-HB2-3**	1.65	1.84	1.84	27	1592
**14-6c-HB2-4**	1.65	1.73	2.02	24	1611
**14-6c-HB2-5**	1.65	1.72	1.70	36	1668
**14-6c-HB2-6**	1.67	1.64	1.74	21	1561
**14-6c-HB2-7**	1.67	1.59	1.72	25	1568
**14-6c-HB2-8**	1.68	1.58	1.91	19	1629
**14-6c-HB2-9**	1.67	1.61	1.94	34	1536
**14-6c-HB2-10**	1.64	1.67	1.72	30	1672
**14-6c-HB2-11**	1.68	1.63	1.74	21	1508
**14-6c-HB2-12**	1.67	1.74	1.77	19	1523
**14-6c-HB2-13**	1.66	1.70	1.88	29	1574
**14-6c-HB2-14**	1.65	1.72	1.78	25	1561
**14-6c-HB2-15**	1.66	1.74	1.78	16	1545
**14-6c-HB2-16**	1.66	1.70	1.85	21	1591
**14-6c-HB2-17**	1.66	1.68	1.89	20	1543
**14-6c-HB2-18**	1.67	1.67	1.82	31	1552
**14-6c-HB2-19**	1.67	1.65	1.70	29	1580
**14-6c-HB2-20**	1.65	1.78	1.86	29	1608
**14-6c-HB2-21**	1.67	1.66	1.91	25	1527
**Range**	1.63 – 1.68	1.58 – 2.09		9 – 53	1500 – 1699

Table S4. Hydration energy of the lowest energy structure reported in Figure 2 and average hydration energy of **6c**, **6c-HB** and **6c-HB2** structure reported in Table S1-S3 for n = 11 – 14.

$Al^+(H_2O)_n$	Water binding energy ΔE_0 / kJ mol^{-1}
11	-54
12	-53
13	-54
14	-51

Cartesian coordinates, harmonic vibration analyses (frequency in cm^{-1}, IR intensity in km mol^{-1}) and energies (E, E_{zpc}, H_{298}, G_{298} in a.u.) of the optimized geometries discussed in the main text.

9-4c

H	2.022691	2.029206	2.369918
H	-2.202665	1.676184	0.640179
H	2.857429	0.225384	-0.640648
H	2.979779	-2.018225	-0.296880
H	-0.089836	-0.258051	-1.508585
H	-1.333988	-1.229881	-1.243331
H	-3.729770	-0.831900	-1.562596
H	-2.892120	0.411060	-1.106445
H	-2.872858	-0.507635	0.882915
H	-2.941419	-0.283028	2.428005
H	-2.459232	2.844395	-0.345938
H	0.154072	-3.235099	0.647890
H	2.238831	1.704147	0.852997
H	3.696108	1.452564	-1.175042
H	-0.709727	-0.341639	1.670587
H	0.789586	0.304364	1.590421
H	-0.319383	1.774921	-1.105845
H	1.194983	1.465271	-1.453352
O	1.595071	1.669044	1.589397
O	2.281148	-1.369492	-0.212670
O	-0.355302	-1.194505	-1.211873
O	-2.990000	-0.555928	-1.012849
O	-2.422061	-0.091408	1.641139
O	-2.103423	1.952512	-0.284401
O	2.876150	1.205839	-0.740348
O	0.234599	-0.524608	1.464197
O	0.269355	1.230806	-1.649533
Al	0.656401	-1.827702	0.207022

29.2, 0.5; 47.7, 1.5; 52.9, 2.6; 66.6, 2.7; 76.7, 0.6; 86.0, 0.4; 98.8, 1.4; 151.5, 3.8; 164.2, 1.8; 175.5, 7.1; 191.0, 1.4; 210.2, 8.2; 229.9, 7.5; 230.4, 2.7; 238.3, 4.4; 249.4, 7.3; 257.9, 11.5; 274.1, 3.5; 280.9, 67.3; 296.4, 56.1; 297.2, 91.2; 321.4, 33.9; 337.5, 31.9; 349.2, 12.4; 356.1, 51.1; 371.5, 19.5; 384.7, 25.0; 423.2, 95.4; 455.4, 116.5; 483.0, 48.0; 493.1, 85.9; 522.3, 58.6; 539.2, 318.0; 568.0, 59.8; 571.4, 120.9; 597.9, 77.0; 603.9, 37.9; 620.2, 10.4; 641.7, 124.5; 656.0, 40.6; 664.0, 287.5; 733.1, 195.2; 758.7, 92.0; 782.0, 288.1; 795.1, 211.2; 836.3, 194.6; 857.3, 152.9; 872.4, 113.7; 924.9, 271.1; 974.1, 52.7; 1033.2, 221.7; 1083.3, 93.2; 1588.8, 120.8; 1601.4, 113.8; 1612.7, 8.2; 1616.1, 13.7; 1617.5, 183.5; 1632.8, 19.9; 1685.8, 71.5; 1736.0, 120.9; 2067.4, 132.5; 2872.9,1174.8; 3089.5,1843.1; 3460.5, 847.5; 3485.0, 683.3; 3571.0,1011.9; 3593.9, 440.7; 3629.8, 168.1; 3668.0, 387.4; 3688.1, 549.7; 3752.7, 416.7; 3804.9, 522.0; 3919.0, 245.0; 3921.7, 207.5; 3927.3, 127.5; 3939.1, 172.8; 3943.8, 153.3; 3982.5, 145.5;

-930.248316111 -930.018854 -929.996721 -930.066737

; ; ;

9-5c

O	0.286047	-0.554756	1.214150
Al	0.313170	-1.477120	-0.325786
O	0.203351	-0.122029	-1.610945
O	-1.653394	-1.633726	-0.315214
O	-1.130524	1.858135	0.854272
O	-2.070926	1.183975	-1.650504
O	1.748860	1.920002	-0.827148
O	2.312375	-1.216637	-0.414896
H	-0.358202	-0.814549	1.878642
H	0.485718	-2.976288	-0.759748
H	0.861568	0.609041	-1.512481
H	-0.677957	0.301398	-1.791356
H	-2.112657	-2.381443	-0.706653
H	-2.253461	-1.119935	0.282722
H	-0.436454	1.173636	0.944203
H	-0.731161	2.697325	1.097535
H	2.264877	1.709398	-0.029451
H	2.254504	2.558776	-1.335312
H	-2.543827	1.715127	-2.294626
H	-1.925157	1.719411	-0.851736
H	2.798945	-0.663737	0.232577
H	2.861833	-1.940212	-0.729262
O	2.660802	0.624930	1.461251
H	3.190797	0.793227	2.244252
H	1.797760	0.239095	1.733220
O	-2.782327	-0.179706	1.576528
H	-3.632519	-0.211050	2.021386
H	-2.509913	0.751837	1.511345

32.2, 1.3; 48.3, 1.9; 60.5, 1.3; 71.2, 1.0; 85.3, 5.1; 109.8, 1.2; 131.2, 1.4; 148.5, 1.0; 169.1, 5.8; 180.4, 5.8; 197.3, 7.6; 212.2, 26.5; 220.1, 7.3; 233.7, 10.1; 244.7, 7.0; 248.5, 59.3; 252.8, 32.7; 255.6, 18.7; 270.1, 100.9; 279.9, 61.6; 287.5, 62.7; 296.6, 26.0; 317.2, 20.8; 320.1, 7.0; 338.4, 59.6; 344.7, 55.0; 361.8, 37.5; 394.6, 31.3; 403.3, 116.3; 426.7, 35.5; 444.4, 191.3; 447.3, 81.9; 464.6, 243.7; 530.8, 99.7; 534.6, 51.0; 558.1, 176.9; 568.5, 157.6; 575.5, 19.1; 609.0, 160.7; 628.9, 106.8; 657.8, 70.5; 677.6, 71.3; 700.6, 55.7; 717.0, 178.5; 735.6, 115.4; 792.9, 218.0; 825.2, 357.1; 884.0, 128.6; 919.1, 81.8; 939.5, 197.9; 1009.1, 249.1; 1049.8, 90.0; 1582.1, 79.4; 1592.8, 78.1; 1610.0, 82.7; 1616.1, 192.7; 1624.7, 47.4; 1638.5, 139.7; 1659.3, 74.5; 1699.8, 91.4; 2005.3, 174.6; 3311.2, 946.2; 3371.2,1024.4; 3419.2, 676.6; 3483.8, 471.3; 3566.2, 819.9; 3592.9, 567.0; 3689.2, 232.4; 3700.7, 494.1; 3722.0, 478.0; 3914.8, 146.7; 3924.0, 207.8; 3928.4, 227.7; 3937.0, 116.9; 3940.0, 220.3; 3946.4, 192.4; 3948.8, 150.8; 3957.2, 188.5;

-930.253134453 -930.023965 -930.001232 -930.071516

9-6c

H	1.935069	-0.995149	2.258260
H	2.690547	-0.007080	1.262353
H	-0.874191	3.409052	0.989372
H	2.772323	-0.860216	-1.110683
H	2.134591	-2.307713	-1.175502
H	-3.864717	-1.223458	0.358081
H	-3.277287	-2.467275	1.049115
H	2.660734	1.192127	-0.429477
H	-1.116444	0.908565	1.401099
H	-1.749118	-0.553684	1.342678
H	-2.229782	1.896513	-0.616334
H	-3.131181	1.628789	-1.860685
H	0.223380	-2.236959	0.600819
H	0.681560	1.037049	-1.457819
H	-0.162291	2.229922	0.197575
H	-1.565101	-1.641023	-1.007752
H	-1.554535	-0.139304	-1.396662
H	4.241856	1.283378	-0.394867
O	1.824131	-0.360277	1.544775
O	-1.004642	2.537463	0.608886
O	1.938782	-1.366423	-1.138221
O	-3.036378	-1.652064	0.597551
O	3.491422	0.702720	-0.247600
O	-0.914112	-0.048863	1.393745
O	-2.608273	1.196731	-1.181275
O	0.826000	0.956338	-0.511757
O	-0.967858	-0.924318	-1.268878
Al	0.445540	-0.729537	0.124048

49.8, 6.6; 64.8, 2.1; 71.5, 1.3; 83.7, 3.2; 117.7, 1.3; 133.5, 4.8; 143.5, 1.8; 157.1, 2.9; 190.6, 3.6; 198.1, 7.3; 211.3, 1.1; 222.6, 1.5; 233.2, 1.8; 254.9, 2.9; 259.1, 14.4; 270.9, 25.8; 281.6, 13.0; 296.6, 9.5; 307.0, 45.6; 312.2, 74.0; 328.7, 127.1; 329.9, 34.0; 336.9, 20.3; 354.0, 31.0; 356.5, 20.1; 368.2, 20.1; 383.1, 44.0; 396.5, 57.7; 412.1, 47.0; 433.1, 51.7; 436.5, 83.9; 456.8, 228.4; 469.2, 209.1; 490.7, 129.1; 499.5, 181.3; 516.8, 79.9; 533.2, 108.2; 583.3, 23.5; 624.3, 33.7; 639.9, 134.7; 672.7, 227.9; 681.6, 125.4; 740.5, 353.8; 771.4, 185.7; 809.2, 123.0; 853.6, 333.7; 861.3, 228.2; 879.9, 161.3; 897.3, 15.6; 936.3, 313.9; 997.0, 127.5; 1012.7, 69.3; 1559.7, 35.9; 1585.9, 191.4; 1608.5, 120.2; 1609.4, 86.6; 1622.3, 59.6; 1632.5, 220.3; 1664.6, 28.4; 1692.1, 126.2; 1893.7, 224.5; 3419.3, 235.1; 3445.3,1118.3; 3543.6, 195.1; 3585.4, 726.1; 3615.3, 169.8; 3633.7, 244.4; 3669.6, 440.7; 3672.7,1363.0; 3803.1, 263.9; 3850.8, 21.6; 3915.2, 163.5; 3925.6, 191.0; 3927.1, 125.3; 3942.6, 113.3; 3943.7, 245.4; 3947.0, 113.9; 3958.2, 163.2;

-930.252795321 -930.021358 -929.999503 -930.066821

10-5c-a

O	0.892249	-0.641775	-1.354330
Al	-0.146149	-1.235427	0.002165
O	-0.009713	0.060273	1.325711
O	1.511094	-2.036711	0.856526
O	3.455562	-0.840271	-0.473234
O	-3.891145	-1.889710	-0.307497
O	2.359787	1.162431	1.280739
O	-1.738171	-0.369603	-0.704345
H	0.550633	-0.838339	-2.229275
H	-0.912339	-2.598367	0.203210
H	-0.724140	0.707184	1.564154
H	0.894093	0.449386	1.498228
H	1.473816	-2.865916	1.339486
H	2.420516	-1.855615	0.536191
H	2.721003	-0.770340	-1.117163
H	4.261354	-1.067583	-0.943593
H	3.042531	0.580415	0.915773
H	2.180939	1.799238	0.572030
H	-4.844229	-1.786686	-0.260245
H	-3.688085	-2.786360	-0.028591
H	-1.832306	0.591069	-0.870437
H	-2.610421	-0.812804	-0.585178
O	-1.519165	2.361734	-0.938290
H	-1.953446	2.933501	-1.577017
H	-0.551563	2.462425	-1.071369
O	-1.922972	1.815499	1.720991
H	-1.957731	2.364491	0.921506
H	-2.140713	2.361270	2.479472
O	1.198217	2.077646	-1.174542
H	1.719857	2.487057	-1.870106
H	1.164232	1.110421	-1.359069

23.0, 0.3; 43.7, 3.9; 48.9, 14.1; 65.1, 12.5; 71.9, 36.0; 76.9, 5.0; 88.8, 4.7; 104.8, 0.8; 109.6, 6.2; 147.7, 39.8; 159.1, 2.7; 163.4, 78.8; 170.9, 2.3; 175.8, 71.0; 193.1, 40.9; 196.4, 1.8; 206.8, 31.8; 219.6, 0.3; 239.4, 13.2; 251.6, 104.2; 257.4, 15.4; 258.4, 15.5; 275.0, 27.2; 283.2, 86.2; 303.5, 4.6; 323.2, 32.2; 326.6, 60.3; 348.0, 83.1; 360.1, 24.2; 367.1, 19.5; 373.4, 56.9; 380.7, 16.4; 392.1, 29.9; 419.4, 40.9; 439.7, 30.5; 454.2, 114.4; 487.1, 101.4; 492.0, 24.0; 528.5, 31.1; 549.1, 139.3; 560.1, 42.2; 595.3, 59.4; 602.6, 18.5; 630.7, 562.2; 636.4, 178.5; 667.0, 203.8; 703.7, 70.8; 716.3, 104.2; 723.8, 330.3; 757.4, 210.9; 841.8, 182.0; 863.6, 107.8; 889.3, 95.8; 893.0, 40.9; 936.1, 340.0; 982.9, 292.8; 988.5, 9.5; 1057.3, 110.5; 1583.7, 126.4; 1585.5, 56.4; 1608.3, 123.3; 1615.7, 84.2; 1632.9, 154.6; 1638.9, 34.1; 1662.3, 61.5; 1668.3, 75.0; 1691.1, 53.5; 1979.0, 199.9; 3211.2,1195.3; 3320.0,1080.7; 3444.6, 516.9; 3469.6,1107.7; 3524.0, 428.5; 3535.3, 958.6; 3593.1, 759.2; 3609.6, 884.9; 3741.8, 292.6; 3753.3, 219.9; 3824.7, 419.4; 3878.4, 38.4; 3927.4, 109.3; 3931.6, 178.4; 3938.7, 222.0; 3941.9, 148.4; 3945.8, 118.7; 3960.2, 191.1; 3992.9, 166.3;

-1006.694386900 -1006.440074 -1006.414329 -1006.492370

10-5c-b
O 0.339830 0.256011 1.568030
Al -0.466748 -0.629072 0.232767
O -0.072476 0.406881 -1.318448
O -2.120403 0.419678 0.388664
O 0.783410 2.896531 0.520894
O -1.451321 2.699894 -0.889728
O 2.496445 1.269049 -1.173382
O 1.213179 -1.565772 -0.190984
H -0.183352 0.440211 2.349878
H -1.292932 -1.976811 0.210558
H 0.881672 0.611265 -1.464900
H -0.582839 1.234605 -1.458530
H -2.988363 -0.045506 0.347371
H -2.195725 1.338391 0.062630
H 0.706040 2.063332 1.019303
H 1.547724 2.760059 -0.054763
H 2.925334 0.836316 -0.408738
H 3.167395 1.390500 -1.850311
H -1.888409 3.433286 -1.329134
H -0.689182 3.048654 -0.364605
H 2.038052 -1.348231 0.282696
H 1.167673 -2.530257 -0.387193
O 2.948464 -0.047731 1.197419
H 3.653964 -0.080880 1.847868
H 2.099975 0.148432 1.657808
O 0.949478 -4.187320 -0.644753
H 1.360277 -4.766067 -1.291448
H 0.178002 -4.647531 -0.305245
O -4.373956 -1.010435 0.227091
H -5.244966 -0.880807 0.610148
H -4.293818 -1.945308 0.022222

26.3, 0.4; 30.6, 4.1; 38.0, 0.8; 43.9, 2.2; 65.0, 0.5; 89.0, 0.9; 100.8, 3.5; 105.5, 7.1; 112.3, 22.9; 125.3, 2.1; 138.9, 4.7; 142.4, 1.7; 168.6, 3.1; 173.1, 154.4; 180.7, 23.9; 186.4, 25.2; 192.6, 227.6; 214.0, 53.7; 219.3, 24.5; 242.0, 24.2; 254.2, 29.9; 258.9, 16.3; 286.8, 16.2; 293.7, 32.3; 300.3, 3.0; 321.4, 57.0; 326.4, 22.0; 330.1, 9.2; 356.6, 20.6; 360.9, 33.9; 369.6, 34.2; 390.4, 41.9; 400.3, 128.3; 414.4, 41.8; 458.8, 148.2; 477.9, 49.0; 497.2, 130.5; 510.6, 121.3; 513.9, 33.1; 531.4, 13.2; 559.8, 99.0; 581.4, 58.5; 593.1, 115.6; 632.5, 33.4; 668.2, 30.0; 678.9, 59.4; 709.6, 49.5; 747.3, 127.9; 761.6, 192.9; 773.4, 338.7; 817.3, 233.6; 854.1, 221.6; 883.1, 330.1; 900.0, 423.2; 959.7, 39.2; 981.8, 195.3; 985.5, 105.2; 1038.1, 86.1; 1603.0, 121.0; 1609.4, 99.9; 1609.8, 35.0; 1611.1, 164.1; 1622.3, 44.8; 1655.9, 234.2; 1659.1, 75.9; 1671.2, 13.1; 1699.8, 76.9; 1960.3, 197.5; 3391.5, 459.6; 3448.1, 653.3; 461.0,1300.1; 3469.7, 964.9; 3484.0, 652.1; 3528.4, 732.8; 3610.6, 379.8; 3628.8, 941.0; 3666.4, 786.0; 3684.9, 605.1; 3837.4, 310.7; 3878.2, 33.2; 3879.9, 49.2; 3934.8, 153.5; 3940.4, 199.1; 3942.4, 124.7; 3959.5, 87.4; 3991.2, 226.3; 3993.0, 99.9;

-1006.694997510 -1006.440285 -1006.414327 -1006.493746

10-6c
H -1.757076 -0.144922 -2.457751
H -1.843329 0.866445 -1.223715
H -0.295882 3.285105 0.020842
H -2.270452 -1.563377 0.758911
H -2.185025 -2.750834 -0.309048
H 2.889224 0.365310 1.068049
H 3.537034 2.249873 -0.450770
H 3.111632 -0.421237 2.399458
H -1.558961 0.093607 1.591253
H 2.010171 0.531874 -1.203424
H 1.716153 -0.410653 -2.460129
H -3.402197 2.491462 -0.435949
H -2.893833 1.412170 0.572677
H -0.169629 -2.155135 -1.947384
H 2.018468 2.311365 -0.028624
H 0.637776 -0.056217 1.286487
H -0.013008 1.796401 0.392132
H 1.549611 -3.058887 -0.168424
H 1.880921 -1.837368 0.788209
H -2.739739 -0.001255 2.644065
O -1.319969 0.143423 -1.652735
O 0.399135 2.675778 0.275111
O -1.697252 -2.050265 0.130906
O 2.784294 1.708360 -0.199972
O 2.603807 -0.413988 1.584368
O -2.536042 -0.018434 1.705490
O 1.497463 -0.238890 -1.540990
O -2.647974 1.942122 -0.209539
O -0.104720 0.027251 0.675232
O 1.171487 -2.309173 0.300033
Al -0.109515 -1.133323 -0.721547

41.3, 5.9; 51.6, 1.9; 67.0, 1.9; 73.1, 1.0; 83.4, 1.1; 115.5, 0.9; 123.8, 3.4; 141.0, 11.1; 151.4, 8.0; 186.3, 12.9; 190.2, 3.6; 205.4, 20.8; 209.7, 27.0; 217.9, 6.7; 224.0, 33.5; 234.7, 10.9; 241.5, 81.7; 244.9, 15.1; 249.9, 15.8; 264.4, 39.5; 269.7, 49.1; 296.7, 4.3; 309.1, 20.2; 319.0, 41.1; 320.6, 39.8; 329.1, 107.3; 336.8, 5.1; 343.1, 106.7; 349.0, 86.4; 367.5, 4.1; 391.3, 27.7; 398.8, 1.5; 415.9, 44.3; 428.8, 53.3; 430.5, 226.8; 454.7, 179.6; 456.0, 83.9; 480.4, 95.7; 534.2, 105.7; 562.1, 55.6; 579.1, 210.6; 590.1, 123.6; 594.6, 30.0; 620.3, 63.2; 636.0, 31.3; 661.3, 60.3; 679.2, 121.2; 723.2, 195.7; 740.9, 65.8; 772.0, 296.4; 809.7, 196.3; 842.8, 150.4; 884.7, 250.3; 892.1, 373.9; 914.0, 99.6; 933.4, 93.9; 956.2, 134.1; 1031.4, 208.0; 1588.3, 89.6; 1601.1, 210.3; 1608.2, 22.4; 1620.5, 50.8; 1635.2, 244.3; 1640.1, 106.5; 1656.6, 70.5; 1661.2, 71.7; 1662.4, 36.3; 1893.2, 239.5; 3356.5, 175.1; 3378.1, 251.0; 3386.2,1759.0; 3472.4, 356.8; 3540.1,1066.9; 3572.1, 582.6; 3599.5, 877.6; 3635.8, 581.5; 3651.2, 576.2; 3832.7, 351.0; 3920.4, 142.5; 3933.3, 85.5; 3933.7, 75.4; 3934.4, 178.5; 3937.2, 279.5; 3938.8, 274.0; 3942.5, 150.0; 3945.2, 124.2; 3964.3, 137.0;

-1006.696462250 -1006.440368 -1006.415758 -1006.489205

; **11-5c**
O -0.404728 0.740289 -1.318445
Al -0.608780 -0.831386 -0.460920
O -0.030421 -0.470848 1.308833
O 1.238825 -1.311209 -0.928141
O 1.785503 1.995446 -0.119370
O 2.613252 0.082364 1.720709
O -4.081344 -2.247940 -0.182624
O -0.869026 1.973821 1.995759
O -2.379365 -0.231535 0.194056
H 0.106825 0.748937 -2.134264
H -1.195659 -2.244594 -0.828200
H -0.456723 0.319383 1.724284
H 0.924477 -0.433343 1.538276
H 1.809551 -1.955150 -0.448382
H 1.788054 -0.740416 -1.504645
H -4.856102 -2.602377 0.259629
H -3.615433 -2.990400 -0.577886
H 0.901316 1.669568 -0.399504
H 1.649827 2.885119 0.218474
H -1.439185 2.333240 1.289975
H -1.216290 2.302159 2.828438
H 2.938248 0.442725 2.550294
H 2.530460 0.835030 1.099052
H -3.097562 -0.903407 0.129288
H -2.719357 0.663836 0.015728
O -2.229985 2.406647 -0.377389
H -2.710200 3.100422 -0.834656
H -1.577101 2.004458 -0.995855
O 2.312633 0.649373 -2.452590
H 2.929492 0.687761 -3.187488
H 2.524928 1.372710 -1.840901
O 2.810800 -2.542502 0.776249
H 3.142176 -1.768922 1.254605
H 3.423232 -3.269976 0.899327

26.3, 3.1; 36.7, 7.5; 42.4, 0.2; 49.9, 0.3; 56.5, 2.5; 63.9, 0.7; 79.1, 1.4; 97.9, 1.0; 106.3, 5.6; 125.0, 1.8; 138.4, 28.2; 148.5, 50.3; 158.0, 2.2; 177.9, 2.6; 184.4, 9.6; 193.5, 29.1; 202.0, 34.1; 211.4, 104.6; 217.7, 9.0; 226.0, 24.7; 235.5, 121.4; 238.0, 31.3; 249.1, 13.3; 256.4, 21.7; 281.4, 101.3; 293.9, 23.0; 300.0, 48.5; 314.0, 63.4; 320.6, 26.5; 327.9, 31.4; 337.7, 60.1; 350.7, 9.4; 356.9, 22.3; 367.4, 14.7; 384.0, 141.0; 403.0, 52.1; 408.7, 157.7; 413.2, 78.4; 442.5, 29.7; 451.0, 100.9; 472.3, 72.0; 496.8, 19.8; 523.9, 48.4; 529.3, 53.7; 537.3, 96.4; 542.3, 109.5; 567.3, 263.1; 589.4, 104.0; 603.7, 21.2; 635.0, 120.9; 662.8, 74.5; 708.5, 49.9; 719.3, 36.2; 740.7, 131.8; 753.6, 376.8; 821.8, 137.7; 842.1, 277.2; 863.6, 346.6; 879.3, 176.0; 922.3, 219.2; 956.6, 334.5; 989.2, 54.6; 1000.1, 29.2; 1027.0, 111.4; 1572.6, 138.7; 1590.7, 138.7; 1598.9, 36.7; 1600.9, 139.6; 1625.1, 70.4; 1636.1, 36.0; 1647.5, 66.9; 1656.5, 138.3; 1668.6, 32.1; 1702.0, 114.2; 1987.7, 209.2; 3406.1, 575.6; 3455.8,1143.7; 3463.5, 937.7; 3466.7, 661.7; 3494.1, 315.0; 3535.5, 818.9; 3563.1, 735.9; 3604.0,1035.2; 3638.4, 391.3; 3690.8, 842.9; 3740.7, 255.0; 3784.6, 221.1; 3869.5, 31.9; 3882.7, 214.4; 3926.2, 92.0; 3930.8, 200.3; 3944.3, 175.0; 3948.8, 197.2; 3950.5, 117.9; 3972.8, 208.5; 3986.5, 168.1;

-1083.134488100 -1082.853819 -1082.825618 -1082.909108

11-6c
H -1.679885 0.611820 2.625738
H -2.515279 -0.325101 1.626989
H 0.388730 -3.697988 -0.563159
H -2.463425 1.652294 -0.761805
H -1.466232 2.804489 -0.312504
H 2.165718 -2.042179 -1.186588
H 3.526453 -0.623814 0.247376
H 3.299162 -1.494010 -2.118680
H -2.577237 -1.419817 -0.049644
H 0.841600 -1.638543 0.967445
H 1.724627 -0.452973 1.495974
H 3.301457 3.219449 0.104531
H 3.467873 1.729803 0.532067
H 0.263835 1.807033 1.286704
H 4.206404 -0.480633 1.644753
H -4.785186 1.239039 -1.296371
H -1.241794 -0.465100 -1.532184
H -0.091704 -2.191203 -0.714878
H 1.754561 1.584665 -0.754063
H 1.598542 0.143578 -1.335109
H -4.112696 0.096946 -0.458824
H -3.782502 -2.256508 0.557189
O -1.785337 0.319892 1.717127
O 0.671254 -2.783172 -0.496405
O -1.515103 1.891375 -0.608934
O 3.480187 -0.157690 1.104299
O 2.774548 -1.290500 -1.339373
O -3.412714 -1.375074 0.472267
O 0.801283 -0.732192 1.324942
O 3.177867 2.313391 -0.185476
O -1.044225 -0.749277 -0.635482
O 1.083274 0.898838 -0.993702
O -3.948282 0.913475 -0.960313
Al -0.314234 0.631248 0.369496

32.7, 1.0; 44.1, 1.7; 46.7, 4.0; 55.5, 5.5; 73.2, 2.5; 90.1, 5.1; 117.3, 14.4; 127.8, 6.5; 133.5, 9.8; 161.2, 25.7; 165.6, 43.5; 173.9, 18.8; 192.8, 16.4; 198.1, 15.7; 203.2, 13.1; 206.9, 62.2; 215.5, 44.8; 222.7, 76.1; 225.4, 10.5;

235.3, 15.2; 250.5, 34.9; 267.6, 16.4; 271.0, 29.2; 282.4, 3.3; 289.8, 34.6; 311.3, 4.3; 323.8, 43.3; 330.2, 36.5; 345.8, 5.0; 348.4, 22.4; 352.1, 12.1; 360.4, 91.0; 363.6, 43.4; 381.0, 17.1; 386.3, 16.4; 401.3, 103.8; 437.0, 190.3; 441.7, 69.5; 451.7, 114.5; 461.3, 14.8; 487.2, 63.8; 510.0, 219.4; 533.9, 32.2; 535.9, 36.4; 545.7, 74.9; 555.6, 192.7; 570.2, 116.6; 603.9, 102.8; 635.7, 32.2; 650.5, 112.7; 655.8, 121.8; 710.5, 225.2; 718.0, 366.3; 777.1, 91.2; 780.5, 152.3; 828.7, 112.7; 868.4, 281.9; 877.5, 398.5; 901.4, 193.6; 917.8, 43.8; 939.7, 217.2; 955.3, 48.7; 976.9, 233.3; 1021.2, 66.3; 1568.0, 25.7; 1591.9, 69.7; 1597.0, 206.0; 1603.6, 171.4; 1606.9, 33.1; 1628.7, 38.6; 1632.9, 144.6; 1660.2, 105.1; 1684.4, 54.3; 1711.1, 153.5; 1883.4, 217.0; 3364.2, 776.0; 3388.1,1242.8; 3409.2, 833.7; 3457.9, 306.6; 3564.0, 216.4; 3577.2, 826.0; 3597.7, 557.6; 3632.9, 340.4; 3675.1, 362.1; 3683.0,1191.9; 3709.4, 514.4; 3764.1, 256.4; 3919.0, 68.5; 3921.0, 126.3; 3928.6, 188.7; 3932.9, 146.8; 3942.8, 243.1; 3944.9, 102.7; 3949.4, 184.9; 3963.8, 157.4; 3965.6, 187.9;

-1083.139706480 -1082.857225 -1082.830176 -1082.909589

11-6c-HB

H 0.454653 -2.423573 -0.237021
H -1.164408 -2.257087 -0.216154
H -1.221441 -0.087727 2.484326
H -2.376200 0.090742 1.375790
H 1.779376 -0.805936 -1.407655
H 2.053075 0.726730 -1.198214
H 3.574332 1.038627 0.394156
H 3.853032 2.368828 -0.380688
H 1.044063 -0.367600 1.306532
H -1.201066 0.277168 -1.889058
H 1.378235 2.219518 0.473344
H -0.168834 2.669040 0.229041
O -0.289158 -1.812520 -0.345464
O -1.410565 0.078876 1.558376
O 1.342800 0.065208 -1.312654
O 3.160601 1.750404 -0.132934
O -1.370970 0.559774 -0.988528
O 0.446380 1.948278 0.506232
Al -0.024622 0.077303 0.155663
O 2.435571 -2.381356 -0.732975
H 2.895083 -3.080977 -1.204849
H 3.048518 -2.032885 -0.058289
H 3.988616 -0.856597 1.983741
H 2.545111 -0.565498 1.439988
H -1.772418 2.350854 -0.892786
H -1.957026 3.915076 -0.930560
O 3.493343 -0.661055 1.182755
O -1.674355 3.179553 -0.384426
H -4.463844 0.486701 0.114352
H -3.003336 0.275841 -0.472945
H -3.360108 -1.874822 0.107615
H -3.262230 -3.407234 -0.175239
O -3.667634 -0.044581 0.185683
O -2.758901 -2.640596 0.105304

41.6, 2.1; 44.5, 1.6; 61.8, 3.9; 69.3, 0.9; 77.1, 4.1; 98.3, 0.4; 124.2, 1.6; 136.8, 3.9; 147.8, 1.5; 156.8, 14.9; 158.7, 4.6; 171.0, 7.7; 180.8, 69.9; 207.6, 10.8; 211.9, 27.8; 216.1, 17.0; 219.1, 37.2; 227.1, 3.4; 231.0, 38.2; 251.0, 0.6; 251.0, 55.6; 262.7, 71.1; 293.1, 2.9; 297.0, 54.1; 317.5, 25.4; 319.1, 18.3; 338.6, 1.7; 351.7, 5.5; 361.4, 27.8; 366.2, 30.0; 369.8, 28.8; 380.3, 32.7; 386.1, 75.0; 389.6, 12.3; 396.0, 71.4; 414.7, 86.2; 431.4, 43.9; 444.6, 97.8; 478.1, 30.0; 488.1, 81.9; 494.6, 196.2; 506.7, 176.3; 523.1, 169.7; 541.1, 50.0; 576.4, 224.5; 599.3, 69.0; 617.1, 23.0; 634.0, 34.1; 640.9, 132.4; 676.3, 164.2; 714.9, 46.5; 726.2, 424.3; 738.1, 187.4; 763.0, 238.1; 778.3, 52.7; 802.4, 159.3; 870.2, 274.0; 883.5, 227.7; 897.7, 123.9; 904.8, 153.7; 960.5, 227.2; 981.5, 116.9; 999.4, 123.6; 1012.2, 296.7; 1572.8, 5.6; 582.6, 60.5; 1589.6, 241.1; 1617.2, 127.1; 1621.6, 264.1; 1621.8, 45.6; 1636.7, 32.6; 1641.8, 128.1; 1648.2, 93.2; 1688.0, 11.8; 1788.7, 569.7; 3366.8, 547.7; 3395.1,1066.6; 3409.2, 789.9; 3426.1, 431.2; 3525.7,1288.0; 3581.2, 259.8; 3594.9, 295.0; 3623.5, 313.1; 3652.1, 818.2; 3664.6, 627.1; 3689.2, 619.1; 3742.8, 674.4; 3787.2, 428.2; 3917.6, 165.4; 3934.3, 202.9; 3937.1, 114.6; 3944.3, 177.4; 3949.4, 177.4; 3952.9, 64.5; 3961.0, 212.4; 3964.5, 129.9;

-1083.140471770 -1082.857432 -1082.831014 -1082.908536

12-6c

H 1.894007 1.899003 -0.923563
H 3.027184 0.744247 -0.663206
H 4.812972 -1.252785 -0.043549
H 2.230675 0.707930 2.593702
H 3.068102 -0.276159 1.681848
H -1.378627 -2.618790 -0.976279
H 0.200135 -2.586016 -2.953917
H -3.242770 -1.277767 -0.312600
H -2.204830 -3.503832 0.010117
H 1.242340 4.217080 -1.263077
H -3.267009 0.574474 1.076303
H -3.150499 1.358654 2.431953
H -0.546213 -0.382175 -1.746411
H -1.062135 1.025793 -1.096222
H 0.283105 1.498830 1.132086
H 0.080116 3.254209 -0.882578
H 0.649570 -2.068682 -1.517400
H -2.472792 3.010238 -0.899021
H 1.286970 -2.222371 0.585049
H 3.238647 -1.351131 -0.140260
H -1.310772 -0.161954 1.828276
H -1.113963 -1.537885 1.099513
H -4.649120 -0.654908 -0.537459
H -1.936717 2.299938 0.375007
O 2.083564 0.961685 -0.678441
O 3.992995 -0.786843 0.133561
O 2.172074 -0.044797 1.997399
O -0.108455 -2.166026 -2.148328
O -1.941974 -2.597669 -0.172529
O -2.656412 1.080927 1.656333
O -0.537713 0.208338 -0.973672
O 0.978606 3.295400 -1.252659
O 1.394430 -1.527699 -0.068826
O -0.637870 -0.832654 1.582820
O -3.758925 -0.454739 -0.241164
O -1.689660 2.600134 -0.522181
Al 0.768949 0.108894 0.511014

40.1, 0.7; 44.5, 1.4; 60.5, 2.0; 68.0, 1.6; 76.1, 1.6; 87.3, 0.7; 90.8, 3.2; 112.8, 0.3; 129.7, 1.6; 136.9, 7.1; 150.9, 1.5; 159.3, 40.0; 168.5, 27.9; 176.8, 46.1; 188.9, 4.2; 193.4, 4.3; 205.3, 11.4; 218.7, 5.0; 224.5, 1.8; 237.4, 15.1; 237.6, 4.6; 245.3, 24.5; 249.8, 56.5; 263.6, 13.4; 273.6, 21.7; 282.5, 5.1; 289.5, 6.6; 311.8, 9.9; 325.3, 14.9; 335.0, 19.4; 350.8, 10.7; 356.3, 57.6; 359.3, 3.5; 362.9, 9.0; 366.5, 27.9; 370.3, 81.6; 377.8, 71.4; 395.4, 16.5; 409.5, 59.2; 430.7, 90.4; 435.6, 173.6; 442.3, 50.1; 459.5, 127.7; 467.4, 78.4; 488.4, 184.4; 501.9, 350.9; 511.1, 65.0; 529.3, 25.3; 533.3, 139.3; 551.7, 86.1; 576.5, 19.0; 605.6, 187.4; 624.7, 204.6; 655.9, 96.8; 668.0, 79.4; 681.9, 84.8; 713.4, 168.9; 727.0, 192.2; 778.0, 194.2; 812.9, 347.1; 816.2, 78.9; 831.3, 79.7; 863.0, 378.2; 882.5, 33.2; 897.5, 134.0; 901.9, 87.7; 922.7, 319.1; 947.6, 218.4; 981.2, 187.1; 1026.1, 63.2; 1571.4, 16.5; 1590.9, 131.5; 1596.8, 124.8; 1605.1, 177.9; 1613.4, 99.8; 1618.1, 81.8; 1630.7, 121.8; 1631.9, 146.2; 1636.6, 18.5; 1643.0, 7.5; 1697.1, 73.4; 1891.6, 201.4; 3355.0, 700.8; 3434.3, 760.6; 3498.7, 560.6; 3535.6, 511.9; 3542.4, 14.7; 3543.6, 201.7; 3580.2,1043.5; 3614.1, 845.9; 3624.0, 294.5; 3644.2, 734.3; 3688.0, 491.0; 3705.4, 491.6; 3724.6, 852.8; 3783.8, 554.2; 3914.9, 177.5; 3929.9, 115.5; 3931.7, 160.1; 3932.5, 71.8; 3935.9, 109.4; 3949.3, 111.0; 3951.7, 196.2; 3955.6, 149.9; 3965.8, 166.6;

-1159.579181870 -1159.270111 -1159.241041 -1159.323998

12-6c-HB

H -0.217978 1.826153 1.410609
H -1.764438 1.450741 1.215248
H -4.239720 1.544521 0.602176
H 1.198718 1.151314 -1.116077
H -0.339500 1.445446 -1.467029
H -2.779488 -3.898817 -0.265063
H -1.445374 -2.460556 1.413888
H -0.469696 -1.812576 2.492300
H 1.158060 -0.373646 1.015032
H -2.403313 -2.364810 -0.394542
H -1.917577 -0.459063 -1.464767
H -3.119340 0.589521 0.005698
H 1.249660 -2.259948 -0.834349
H -0.275756 -2.807425 -0.773580
O -0.867671 1.087463 1.354056
O -3.330966 1.508943 0.296433
O 0.303885 0.759167 -1.166594
O -2.096568 -3.247907 -0.090080
O -1.005873 -1.638730 1.714582
O -1.948051 -0.605466 -0.515519
O 0.294844 -2.023474 -0.756263
Al -0.289016 -0.435346 0.266579
O 1.019507 2.982103 1.201707
H 1.756719 2.794504 0.596949
H 1.286575 3.700502 1.777777
H -1.864800 3.146437 -2.319265
H -2.448176 2.336807 -1.114794
H 3.439340 -1.723660 -0.336111
H 3.504590 -2.730925 -1.529129
O -1.729599 2.371209 -1.771708
O 2.929149 -2.369726 -0.852583
H 2.695504 -0.317303 1.128273
H 4.193853 -0.257275 1.595936
H 3.324268 1.200033 -0.215149
H 3.352892 2.290852 -1.337016
O 3.627380 -0.245518 0.819336
O 2.789235 1.878018 -0.677434

19.9, 0.8; 36.3, 3.1; 37.8, 1.7; 47.4, 0.1; 65.3, 0.3; 78.4, 3.6; 87.9, 5.0; 112.8, 2.3; 128.2, 20.0; 136.1, 21.1; 148.7, 11.7; 150.7, 139.1; 162.4, 11.5; 167.2, 12.3; 172.0, 82.3; 177.9, 10.9; 189.1, 68.1; 205.0, 12.6; 210.8, 5.5; 216.5, 2.0; 229.1, 2.4; 236.2, 12.0; 247.7, 8.1; 253.8, 24.2; 260.0, 13.8; 263.4, 38.2; 288.0, 60.0; 295.7, 22.6; 310.2, 15.8; 317.4, 8.6; 334.8, 23.7; 349.9, 12.4; 355.1, 9.3; 362.3, 34.7; 364.7, 16.6; 376.3, 28.8; 383.4, 117.4; 394.5, 9.4; 399.8, 156.6; 405.1, 21.9; 440.8, 119.2; 453.8, 123.2; 462.6, 13.5; 472.3, 38.1; 487.8, 20.9; 500.3, 212.5; 505.3, 410.2; 523.6, 29.5; 569.9, 41.6; 577.2, 55.1; 594.8, 36.7; 623.5, 74.6; 630.9, 241.3; 636.3, 199.3; 643.3, 117.4; 668.1, 72.0; 704.6, 290.3; 749.1, 79.4; 773.4, 301.1; 789.9, 129.8; 806.2, 86.2; 826.5, 81.4; 846.8, 145.8; 875.8, 155.8; 892.9, 451.4; 906.0, 124.8; 962.7, 15.0; 968.1, 292.8; 999.8, 148.0; 1044.9, 90.5; 1562.9, 25.2; 1574.2, 73.4; 1585.9, 173.4; 1612.1, 49.6; 1619.5, 86.6; 1621.7, 133.2; 1625.7, 151.1; 1636.6, 28.9; 1644.3, 160.7; 1667.7, 80.0; 1687.5, 84.9; 1826.0, 569.1; 3413.4, 154.9; 3427.6,1058.0; 3437.8, 868.0; 3456.9, 150.5; 3477.2,1290.9; 3505.7, 142.5; 3566.8,1535.6; 3593.0, 661.1; 3610.3, 474.9; 3617.8, 824.3; 3683.4, 650.5; 3713.4, 482.1; 3719.3, 383.6; 3765.8, 529.3; 3919.8, 63.3; 3925.4, 165.3; 3934.1, 141.4; 3938.1, 209.0; 3949.8, 118.4; 3951.7, 173.6; 3964.6, 337.1; 3965.1, 135.8; 3965.6, 38.5;

-1159.581559600 -1159.273820 -1159.244202 -1159.330047

12-6c-HB2

H -1.974982 -0.506527 1.687781
H -1.734023 -1.888269 0.919974
H 2.989966 -2.680066 1.764165
H -0.445127 2.810379 -0.712118
H -1.797623 -1.425501 -1.516502

H -2.352660 0.034991 -1.336636
H 3.530350 -1.048900 -0.214475
H 4.363178 -0.384757 -1.359866
H -4.623208 0.936412 -1.083876
H -0.749128 -3.022090 -0.513835
H 1.794354 -0.437672 1.589510
H 1.520739 1.125542 1.354377
H -2.010199 1.575992 1.490842
H -3.015586 1.597441 2.690920
H -0.837822 1.312689 0.189692
H -3.755320 1.024968 0.211189
H 1.059361 -1.863187 -1.260380
H 1.916741 -2.234457 0.683632
H 1.037788 1.578703 -1.568410
H 2.044854 0.344694 -1.342900
H 0.015915 3.945676 -1.685496
H -1.978441 -4.017284 -0.611014
O -1.312561 -1.068598 1.243740
O 2.705532 -1.954196 1.204618
O -1.528482 -0.491488 -1.462204
O 3.472904 -0.522062 -1.031148
O -1.728029 -3.098664 -0.492262
O 1.112437 0.228692 1.371875
O -2.791995 1.140098 1.875365
O -3.726146 0.882952 -0.748328
O 0.330252 3.237173 -1.117038
O 0.597744 -1.814967 -0.419092
O 1.098555 0.621266 -1.400856
Al -0.135821 -0.161502 -0.086240
O 2.094183 2.691128 0.968508
H 1.602416 3.183640 0.291431
H 2.568975 3.326432 1.507702

29.2, 1.6; 36.1, 0.2; 41.6, 1.4; 50.2, 0.5; 79.5, 1.9; 83.0, 2.9; 92.9, 3.5; 111.4, 10.0; 115.2, 24.1; 134.8, 1.9; 141.4, 6.9; 161.0, 74.0; 167.8, 12.5; 174.3, 60.0; 175.3, 3.7; 191.8, 14.2; 202.6, 46.6; 206.3, 17.3; 213.1, 27.7; 216.1, 71.8; 226.9, 5.2; 236.1, 16.2; 243.9, 6.1; 254.9, 11.8; 258.7, 13.0; 269.7, 48.7; 298.5, 5.1; 306.7, 46.8; 315.3, 25.5; 322.5, 70.9; 333.4, 35.1; 338.8, 0.7; 349.7, 6.7; 354.6, 7.5; 365.0, 28.4; 372.7, 116.5; 377.6, 48.5; 383.6, 109.3; 389.1, 35.2; 441.0, 74.9; 448.7, 110.2; 456.4, 32.2; 463.8, 152.1; 471.6, 9.2; 500.7, 595.6; 513.1, 10.3; 522.4, 73.5; 526.5, 74.4; 533.1, 67.3; 568.5, 53.0; 583.1, 72.2; 589.5, 44.7; 618.0, 131.5; 631.5, 40.4; 653.3, 139.6; 670.1, 132.9; 691.9, 160.0; 727.6, 366.2; 759.2, 272.7; 775.3, 163.3; 832.0, 38.8; 859.4, 127.8; 882.2, 327.3; 898.5, 187.4; 915.4, 141.2; 962.8, 240.9; 970.0, 93.9; 1011.2, 143.8; 1038.5, 146.2; 1070.0, 243.3; 1556.4, 41.0; 1559.2, 86.5; 1564.8, 290.3; 1582.7, 160.7; 1610.0, 132.4; 1615.0, 89.4; 1623.7, 88.3; 1644.8, 112.0; 1655.5, 161.6; 1671.4, 44.6; 1677.9, 194.9; 1689.9, 50.9; 3426.6, 400.0; 449.6,1334.6; 3465.6, 740.7; 3472.0, 537.0; 3525.9, 185.1; 3605.2, 651.3; 3609.9, 317.2; 3646.3, 315.0; 3657.8, 500.1; 3681.2, 436.0; 3692.0, 694.8; 3695.3, 293.3; 3712.1, 892.9; 3743.1, 493.3; 3746.4, 454.9; 3921.8, 74.8; 3931.1, 220.6; 3934.8, 173.2; 3948.2, 134.2; 3951.6, 173.0; 3961.8, 228.7; 3963.1, 137.0; 3963.4, 165.3;

-1159.579992560 -1159.271613 -1159.242160 -1159.327004

13-6c
H 0.752855 1.668551 1.630710
H -0.417072 2.552934 0.985031
H -1.765867 4.257723 -0.579636
H 1.370260 1.600013 -1.412853
H -0.002685 2.396393 -1.497607
H -3.609544 -1.567950 -0.356636
H -4.355812 -0.116825 1.714487
H -3.870981 -2.384940 -1.660739
H -1.912762 -0.949413 1.640019
H -0.343581 -1.283084 1.924829
H 1.140935 -0.283517 0.114565
H -3.227973 0.288222 0.677930
H -2.435418 0.566876 -1.280868
H -1.890210 2.680350 -0.525119
H 0.006590 -1.963372 -1.208335
H -1.525645 -1.621975 -1.462252
O -0.149198 1.654766 1.252358
O -1.273172 3.443401 -0.459596
O 0.399029 1.509961 -1.512008
O -3.622006 -0.453167 1.195863
O -3.249872 -1.792169 -1.233014
O -1.010232 -1.000472 1.272938
O -2.026999 0.886465 -0.471519
O -0.616014 -1.248525 -1.444564
Al -0.345641 0.226971 -0.163194
O 2.475122 1.096947 1.899923
H 2.807458 1.174346 0.979357
H 3.117018 1.534496 2.466098
H 1.881412 -0.724923 2.169740
H 1.814915 -1.941906 3.138399
H 1.436521 -2.502521 0.599441
H 1.496255 -3.824878 -0.218293
O 1.451887 -1.590886 2.320537
O 1.389866 -2.873480 -0.303119
H 2.631517 -1.816082 -1.243055
H 3.567831 -1.572570 -2.463839
H 3.208987 0.446154 -1.160169
H 3.812620 1.887604 -1.176854
O 3.250946 -1.183489 -1.646990
O 3.081358 1.369356 -0.829913

30.3, 0.7; 38.2, 1.3; 48.7, 0.5; 69.8, 2.9; 80.1, 16.1; 83.1, 1.8; 98.3, 2.0; 107.6, 4.8; 117.5, 8.9; 129.4, 76.4; 133.5, 15.1; 142.6, 1.8; 151.3, 1.8; 160.4, 8.5; 170.2, 0.5; 181.8, 3.3; 194.7, 6.6; 198.0, 4.5; 204.2, 6.4; 209.1, 4.4; 218.2, 14.8; 226.9, 3.2; 232.7, 9.8; 242.4, 4.5; 244.9, 64.0; 249.5, 30.9; 267.4, 9.7; 276.2, 13.6; 294.1, 7.2; 298.0, 4.4; 311.3, 6.4; 329.1, 12.1; 338.4, 6.3; 349.8, 4.7; 353.1, 1.2; 364.3, 23.1; 368.6, 68.5; 383.8, 36.2; 385.9, 55.2; 408.8, 60.0; 409.2, 20.1; 427.5, 85.9; 430.7, 19.3; 446.3, 29.5; 449.3, 54.1; 466.2, 150.0; 480.6, 187.6; 504.9, 196.1; 510.0, 14.1; 514.0, 134.9; 529.0, 121.4; 544.3, 68.0; 549.6, 52.5; 560.2, 220.9; 583.4, 65.6; 601.3, 69.2; 606.2, 183.9; 619.7, 297.2; 648.7, 109.3; 666.6, 81.6; 685.3, 317.3; 719.3, 71.5; 747.8, 149.3; 782.6, 44.2; 791.9, 274.8; 842.8, 10.3; 848.3, 76.5; 863.2, 24.1; 883.7, 258.5; 889.4, 365.8; 928.9, 358.9; 938.1, 125.2; 977.9, 315.3; 1008.6, 219.7; 1014.1, 65.3; 1036.4, 60.8; 1567.5, 11.2; 1587.5, 150.6; 1594.4, 57.0; 1613.5, 123.9; 1616.6, 21.7; 1622.2, 129.6; 1626.6, 196.4; 1632.6, 44.4; 1637.2, 16.1; 1645.0, 146.2; 1668.0, 93.7; 1673.7, 72.0; 1929.3, 220.8; 3409.0, 780.2; 3437.2, 603.4; 3503.9, 445.6; 3509.6, 488.5; 3520.8, 319.2; 3576.5, 115.9; 3588.8, 872.8; 3607.0, 309.6; 3622.4, 329.1; 3632.4, 442.8; 3649.4,1179.1; 3683.0, 297.8; 3689.7, 625.3; 3705.9, 425.8; 3716.4, 390.6; 3721.6,1420.6; 3914.2, 95.0; 3922.9, 34.8; 3926.1, 127.9; 3928.0, 175.5; 3930.9, 132.2; 3950.3, 134.5; 3953.8, 175.7; 3959.0, 140.6; 3964.9, 157.7;

-1236.025068350 -1235.688728 -1235.657580 -1235.745180

13-6c-HB
H 1.175496 1.601708 1.487909
H -0.141860 2.396600 1.074369
H -1.734682 4.178131 0.132668
H 1.937891 0.055384 -0.954235
H 1.045616 1.289680 -1.442230
H -3.835494 -1.739268 -0.172923
H -4.728665 0.607020 0.488509
H -3.821282 -3.204335 -0.706014
H -2.654672 -0.424555 1.496940
H -1.526725 -1.217188 2.300610
H 0.758283 -0.933333 1.163849
H -3.260196 0.553062 -0.111503
H -1.597066 0.772924 -1.512212
H -1.544270 2.666938 -0.319276
H -0.099798 -2.231433 -1.056166
H -1.669293 -2.070590 -0.678701
O 0.205228 1.521365 1.332322
O -1.083969 3.496374 -0.045815
O 1.020700 0.370258 -1.080415
O -3.960318 0.045354 0.367482
O -3.282983 -2.532058 -0.283768
O -1.693361 -0.526654 1.655017
O -1.615809 0.941488 -0.566240
O -0.813282 -1.603459 -0.804787
Al -0.309898 -0.059498 0.286716
O 2.877174 1.653909 1.415365
H 3.331163 1.001546 0.855226
H 3.493906 1.936130 2.093040
H 1.427967 3.504670 -2.342565
H 0.245967 3.414859 -1.321006
H 1.929258 -3.211231 -0.520038
H 1.563867 -3.771001 -1.931410
O 0.837494 2.896603 -1.895080
O 1.378608 -3.066825 -1.307381
H 1.889500 -1.964327 1.305457
H 2.969441 -2.964727 1.861584
H 3.506728 -1.244268 0.113614
H 4.325655 -0.480419 -0.977179
O 2.664326 -2.523627 1.064558
O 3.596436 -0.391292 -0.358210

25.1, 3.2; 33.9, 1.1; 38.9, 3.0; 49.7, 0.4; 60.7, 0.7; 61.9, 2.2; 73.8, 0.6; 77.6, 1.2; 98.1, 1.3; 122.5, 4.1; 131.7, 24.1; 143.3, 77.4; 151.7, 21.7; 153.8, 43.3; 162.9, 14.8; 166.4, 0.1; 178.5, 32.3; 186.2, 40.3; 192.9, 109.9; 205.7, 9.8; 206.7, 24.9; 218.1, 10.0; 220.8, 42.8; 228.1, 1.4; 236.9, 28.4; 241.9, 1.2; 251.0, 14.5; 255.0, 121.4; 260.4, 70.4; 267.8, 23.4; 288.4, 9.8; 303.0, 12.6; 314.0, 42.1; 331.2, 25.0; 337.4, 2.4; 343.7, 23.6; 345.9, 15.1; 352.4, 14.9; 354.4, 19.0; 363.0, 32.6; 376.0, 33.7; 394.1, 3.2; 399.9, 32.5; 410.4, 121.1; 429.4, 17.6; 452.7, 137.7; 461.1, 68.0; 478.3, 163.6; 484.4, 81.6; 506.8, 150.1; 518.8, 143.7; 537.7, 85.6; 557.4, 45.9; 566.6, 91.0; 578.4, 147.3; 580.7, 43.2; 603.8, 147.9; 621.7, 40.3; 634.0, 132.8; 646.6, 298.9; 662.3, 178.9; 723.4, 79.2; 740.6, 68.7; 757.0, 8.6; 781.4, 441.2; 795.8, 31.2; 805.1, 167.9; 829.1, 121.1; 864.7, 126.0; 887.3, 364.9; 906.2, 383.2; 931.7, 86.4; 984.0, 20.2; 993.8, 217.2; 1013.1, 196.7; 1046.7, 97.9; 1566.8, 28.2; 1581.2, 69.1; 1596.1, 165.0; 1604.3, 30.5; 1614.8, 178.5; 1617.7, 58.8; 1622.6, 146.2; 1636.7, 50.0; 1640.6, 105.0; 1674.9, 66.1; 1678.8, 89.6; 1701.0, 93.8; 1801.7, 579.3; 3381.1, 562.9; 3408.2,1098.6; 3433.2, 132.2; 3442.1, 684.5; 3461.6, 936.3; 3487.9, 124.5; 3515.2,1541.7; 3574.3,1829.1; 3580.4, 636.4; 3618.9, 515.5; 3635.0, 661.9; 3692.1, 663.5; 3706.8, 485.8; 3712.2, 256.1; 3721.2, 418.9; 3921.5, 64.8; 3933.7, 182.1; 3937.3, 123.8; 3945.8, 214.8; 3950.5, 53.0; 3953.8, 226.5; 3960.5, 142.9; 3963.5, 293.7; 3964.9, 61.4; 3968.6, 158.6;

-1236.023872950 -1235.690796 -1235.658382 -1235.750177

13-6c-HB2
H -1.729896 0.705769 1.905304
H -1.986312 -0.836331 1.498866
H 0.741841 2.800209 -0.942291
H -1.969570 -0.530061 -1.578247
H -2.077422 1.004689 -1.124663
H 3.149190 -2.120709 -0.244488
H 2.199256 -3.336958 1.830978
H 4.145467 -1.702648 -1.377421
H -3.843620 2.705923 -0.874792
H -2.992019 -2.385444 -0.939682
H 1.861270 -0.804618 1.573205
H 2.125038 0.729983 1.212747
H -0.933365 2.566194 1.448932
H -1.728100 3.151930 2.665475
H -2.566988 -3.259424 1.444983

H -1.473093 -2.507357 0.581975
H -0.043354 1.726158 0.207631
H -2.870801 2.620097 0.342450
H 1.264247 -2.561547 0.808918
H 0.015593 -2.092380 -0.972235
H 1.550287 1.024004 -1.741872
H 2.085723 -0.443038 -1.369133
H 1.454376 3.593269 -2.089384
H -3.399526 -2.319776 -2.450472
O -1.341688 -0.101488 1.522469
O -1.460503 0.251151 -1.260945
O 2.146953 -2.575613 1.249705
O 3.236467 -1.632347 -1.081512
O -2.746260 -2.030737 -1.810708
O 1.431119 0.046873 1.362795
O -1.790566 2.555592 1.914361
O -2.417302 -2.502847 0.873404
O -2.997746 2.360204 -0.584007
O 1.562713 2.873426 -1.462081
O -0.031097 -1.768593 -0.068249
O 1.291579 0.144200 -1.416462
Al -0.026457 0.073614 0.042080
O 3.194963 1.930783 0.600317
H 2.852711 2.482706 -0.122005
H 3.883957 2.427532 1.045473

20.1, 0.7; 32.3, 0.4; 43.5, 1.8; 46.2, 4.8; 52.1, 1.4; 69.3, 3.1; 83.2, 3.4; 84.2, 7.2; 94.3, 4.0; 114.8, 24.5; 127.7, 10.8; 131.5, 90.8; 146.3, 40.1; 158.4, 8.9; 163.9, 0.9; 170.1, 6.3; 181.0, 73.1; 183.2, 42.9; 197.1, 7.5; 209.0, 67.2; 215.8, 30.6; 220.2, 3.2; 226.2, 0.4; 229.9, 1.9; 236.9, 9.4; 243.2, 101.1; 249.4, 12.3; 257.2, 42.9; 260.9, 6.7; 281.1, 27.9; 297.4, 2.7; 319.0, 20.8; 323.4, 85.1; 330.9, 42.2; 339.9, 31.8; 342.0, 29.0; 345.8, 25.7; 349.6, 4.8; 353.2, 3.3; 370.3, 51.0; 375.6, 71.3; 387.1, 98.4; 399.8, 53.0; 408.1, 22.0; 440.7, 91.5; 453.6, 144.8; 463.4, 117.7; 474.7, 37.2; 479.9, 148.0; 516.4, 420.6; 530.1, 38.4; 534.9, 109.1; 552.7, 96.2; 561.5, 87.3; 574.1, 54.0; 578.7, 29.4; 593.1, 52.4; 622.0, 65.1; 635.9, 56.1; 641.1, 27.9; 672.7, 410.6; 680.6, 197.6; 696.9, 153.9; 710.4, 145.2; 759.5, 284.9; 802.0, 158.1; 853.8, 10.4; 865.3, 28.4; 889.5, 460.9; 891.5, 128.9; 949.0, 127.5; 968.4, 350.4; 1006.4, 126.2; 1019.3, 180.6; 1053.5, 46.2; 1077.7, 244.3; 1558.4, 83.3; 1563.6, 12.7; 1565.1, 339.2; 1593.3, 101.5; 1601.8, 161.6; 1610.2, 143.0; 1616.0, 76.9; 1621.7, 102.6; 1635.7, 100.2; 1653.2, 180.8; 1677.8, 63.6; 1679.7, 117.7; 1691.0, 34.1; 3389.8, 302.4; 3404.9,1412.3; 3449.1, 481.3; 3470.3,1421.8; 3474.1, 89.9; 3509.6, 855.8; 3595.8, 387.7; 3624.0, 743.0; 3631.5, 564.3; 3653.3, 276.0; 3686.9, 464.4; 3693.7, 836.8; 3700.6, 431.1; 3716.0, 524.4; 3739.6, 532.3; 3744.4, 484.0; 3916.4, 76.2; 3933.6, 207.2; 3937.1, 176.2; 3942.9, 109.1; 3952.5, 173.7; 3963.8, 231.7; 3964.1, 77.4; 3965.5, 206.7; 3968.7, 165.6;

-1236.021698670 -1235.688366 -1235.656028 -1235.747806

14-6c
H 0.614265 1.830554 1.524853
H -0.780477 2.501550 1.189404
H -2.421516 4.197641 -0.063583
H 0.827724 1.904375 -1.550821
H -0.580266 2.608790 -1.299461
H -3.746740 -1.743567 -0.285970
H -4.336015 -0.679578 2.039402
H -4.083767 -2.379185 -1.673057
H -1.870151 -1.260398 1.591035
H -0.286647 -1.681035 1.549251
H 1.985034 -1.893526 1.696902
H 1.433916 -3.248186 2.273258
H 0.974395 -0.106063 -0.075526
H -3.364592 -0.032710 0.967377
H -2.798427 0.560723 -0.982718
H -2.377256 2.614869 -0.126162
H -0.206337 -1.691827 -1.574566
H -1.780064 -1.453511 -1.560375
O -0.331620 1.649849 1.340879
O -1.837747 3.436598 -0.072413
O -0.134416 1.757687 -1.455372
O -3.658172 -0.875684 1.389253
O -3.450335 -1.827831 -1.209896
O -0.988274 -1.085346 1.215533
O 1.251489 -2.564217 1.623673
O -2.340852 0.831480 -0.181761
O -0.904545 -1.008018 -1.573192
Al -0.577765 0.304046 -0.122653
O 2.387833 2.013795 1.418642
H 2.594726 2.095546 0.466071
H 2.897269 2.684420 1.881494
H 3.457760 -0.783454 0.604856
H 2.916813 0.138907 1.700256
H 1.214910 -2.844256 -0.157226
H 1.305137 -3.644396 -1.494301
O 3.212068 -0.771999 1.543396
O 1.224690 -2.756086 -1.135946
H 2.571819 -1.492899 -1.399064
H 4.036611 -1.260010 -1.867066
H 2.878629 0.862744 -1.438793
H 3.228718 2.295911 -1.943551
O 3.325320 -0.870551 -1.351317
O 2.645244 1.813570 -1.351157

32.5, 0.8; 45.9, 0.5; 57.8, 3.4; 59.2, 4.8; 77.5, 1.7; 80.3, 3.0; 86.3, 1.6; 91.3, 0.1; 109.3, 0.6; 125.9, 3.3; 133.0, 1.9; 136.2, 1.2; 164.6, 0.3; 171.7, 2.1; 173.8, 6.0; 180.4, 1.4; 186.8, 7.2; 196.6, 5.3; 201.8, 1.6; 212.7, 6.7; 216.6, 3.4; 223.7, 9.4; 231.9, 1.0; 236.6, 41.8; 240.4, 28.8; 244.7, 46.4; 258.1, 10.7; 269.4, 5.4; 271.1, 10.2; 285.7, 14.6; 290.6, 11.7; 298.3, 12.6; 307.3, 1.4; 321.9, 13.7; 334.5, 1.6; 346.6, 12.0; 354.9, 4.1; 358.2, 8.5; 368.7, 21.8; 377.9, 51.6; 382.2, 123.0; 388.7, 65.0; 411.1, 26.4; 424.0, 12.7; 431.0, 92.5; 443.5, 71.4; 446.9, 42.3; 461.6, 35.5; 481.0, 24.6; 492.1, 158.0; 500.5, 140.3; 512.7, 199.9; 516.7, 63.4; 535.8, 55.4; 542.4, 120.0; 551.5, 25.2; 565.8, 107.7; 580.3, 87.1; 588.3, 42.3; 591.0, 49.8; 613.5, 31.2; 619.2, 140.7; 637.4, 233.3; 674.4, 152.0; 695.5, 179.1; 702.3, 245.6; 728.8, 293.1; 746.0, 399.5; 771.9, 104.5; 812.1, 111.5; 836.3, 106.1; 842.3, 11.4; 858.7, 41.5; 871.9, 263.0; 893.7, 297.8; 900.1, 372.4; 943.5, 217.1; 954.8, 195.3; 1001.4, 19.4; 1004.9, 261.9; 1027.6, 49.4; 1083.3, 113.7; 1567.4, 21.7; 1590.0, 143.4; 1614.4, 32.1; 1615.7, 108.0; 1623.5, 124.5; 1630.0, 40.6; 1633.5, 21.4; 1637.7, 116.2; 1642.4, 296.4; 1648.6, 39.5; 1671.9, 98.9; 1676.1, 38.2; 1686.1, 45.7; 1894.2, 210.1; 3256.6, 931.9; 3422.0, 659.0; 3486.9, 193.0; 3495.6, 389.7; 3512.8, 613.2; 3524.8, 609.2; 3544.2, 392.4; 3581.1, 759.8; 3595.5, 151.4; 3606.2,1677.5; 3633.1, 307.7; 3651.5, 445.0; 3665.0, 767.8; 3677.2, 444.9; 3699.9, 602.7; 3721.5,1180.2; 3732.6, 149.0; 3793.1, 521.7; 3911.0, 91.0; 3924.4, 89.2; 3925.0, 86.7; 3930.2, 199.4; 3931.9, 170.0; 3933.1, 97.2; 3949.6, 136.9; 3954.9, 158.7; 3961.0, 147.8;

-1312.470132290 -1312.106584 -1312.073830 -1312.164300

14-6c-HB
H -0.158443 2.033930 1.476685
H -1.721773 1.674050 1.360362
H -4.280335 1.696813 0.960203
H 0.838031 1.731493 -1.354536
H -0.718392 2.075668 -1.367263
H -1.950781 -3.543643 -0.805508
H -3.676534 -2.800757 0.951977
H -1.517504 -4.145101 -2.183209
H -1.265954 -2.121872 1.395724
H 0.224960 -1.778960 1.934273
H -2.293840 3.853256 -1.827336
H -2.663980 2.957154 -0.604106
H 1.268215 0.154497 0.517575
H -2.796148 -1.746880 0.155026
H -2.147142 -0.295329 -1.415627
H -3.158682 0.901622 0.166194
H 1.219784 -1.750289 -1.064334
H -0.248909 -2.189899 -1.441799
O -0.819011 1.308486 1.357843
O -3.401352 1.762441 0.582120
O -0.061733 1.340573 -1.335348
O -2.813372 -2.647142 0.562444
O -1.237167 -3.573423 -1.466539
O -0.538664 -1.472045 1.416715
O -1.931067 3.254328 -1.172271
O -2.041720 -0.301738 -0.461008
O 0.323313 -1.409550 -1.254024
Al -0.277003 -0.043338 0.038938
O 1.171871 3.076268 1.406322
H 1.735267 2.956629 0.620547
H 1.216349 4.001108 1.654842
H 2.584209 -1.396599 2.986947
H 1.968435 -0.651953 1.748604
H 2.680579 -2.311631 0.621346
H 3.164199 -3.180519 -0.585607
O 2.089910 -1.528820 2.173327
O 2.806192 -2.320875 -0.348645
H 3.709793 -0.693315 -0.600400
H 5.011339 0.146504 -0.704460
H 3.066265 1.595633 -0.833404
H 2.908509 2.936024 -1.612974
O 4.066388 0.210271 -0.553681
O 2.461851 2.358197 -0.989247

19.6, 0.5; 32.1, 2.8; 36.0, 0.6; 42.6, 1.0; 47.3, 3.4; 58.8, 2.0; 60.2, 0.7; 77.2, 1.9; 84.6, 1.2; 93.9, 4.1; 117.9, 5.7; 128.0, 8.2; 150.4, 6.2; 152.0, 3.0; 158.2, 18.8; 166.1, 12.7; 174.9, 5.3; 181.8, 31.0; 187.5, 29.1; 196.8, 118.0; 201.3, 64.4; 208.7, 74.4; 221.0, 10.7; 224.4, 26.7; 225.3, 48.2; 229.6, 7.8; 240.4, 8.0; 244.6, 15.5; 249.9, 37.1; 259.4, 16.3; 259.7, 10.3; 277.5, 50.2; 298.1, 5.7; 303.4, 15.6; 316.2, 9.4; 328.0, 23.7; 330.5, 51.6; 340.4, 14.2; 346.6, 4.5; 348.6, 7.1; 357.1, 10.6; 361.7, 50.9; 374.8, 20.7; 384.1, 14.3; 388.8, 55.1; 403.2, 95.8; 423.6, 9.4; 440.3, 83.5; 452.8, 52.5; 463.7, 114.3; 467.0, 190.7; 478.7, 72.6; 479.7, 29.9; 491.2, 71.4; 494.5, 79.3; 518.6, 54.8; 546.0, 41.4; 552.8, 193.3; 572.7, 42.9; 595.1, 292.4; 610.5, 20.7; 613.9, 230.0; 638.3, 47.7; 642.1, 113.9; 652.0, 410.2; 666.8, 42.6; 691.7, 181.7; 727.7, 61.8; 765.3, 206.1; 792.1, 129.9; 807.1, 222.0; 843.5, 81.6; 855.7, 222.6; 878.2, 27.9; 884.5, 430.2; 904.6, 173.2; 937.7, 96.0; 968.7, 275.8; 997.9, 161.5; 1014.0, 138.5; 1034.7, 7.4; 1067.4, 202.9; 1564.3, 103.1; 1567.2, 23.9; 1600.4, 162.6; 1609.6, 25.7; 1618.0, 83.1; 1619.1, 22.2; 1624.9, 104.4; 1629.2, 141.5; 1632.5, 116.3; 1648.9, 54.2; 1670.7, 74.5; 1689.5, 169.1; 1700.7, 52.1; 1782.8, 316.6; 3390.1, 708.9; 3410.8, 658.0; 3440.5, 328.8; 3454.7,1315.1; 3464.6,1206.7; 3473.2, 695.3; 3517.9, 273.0; 3558.6, 750.9; 3589.1, 532.0; 3628.1, 443.4; 3631.8, 925.0; 3661.2, 388.9; 3680.8, 653.4; 3693.9, 370.2; 3703.2, 629.9; 3711.7, 334.6; 3734.3,1115.9; 3929.1, 169.1; 3929.4, 65.6; 3934.7, 149.6; 3936.0, 91.3; 3950.9, 79.8; 3955.3, 235.0; 3959.6, 143.9; 3963.1, 138.6; 3964.7, 176.7; 3966.5, 147.3;

-1312.465099110 -1312.105030 -1312.070527 -1312.167218

14-6c-HB2
H -1.670717 0.701295 1.757773
H -2.030885 -0.722797 1.085182
H -0.958777 -3.580267 1.543059
H 1.097068 3.168207 -0.489563
H -1.943536 0.209861 -1.833750
H -2.042288 1.609356 -1.090328
H 2.767668 -2.144076 -0.506704
H 2.942565 -2.986246 1.857790
H 3.228284 -2.223527 -1.995811
H -3.713519 3.300399 -0.455886
H -1.506875 -1.720112 -1.688721
H 1.737762 -0.919559 1.299561

H 2.171552 0.608756 1.158259
H -0.768664 2.582315 1.684049
H -1.498856 2.949431 3.020862
H -3.921488 -2.172117 0.992667
H -3.104891 -1.997439 -0.324095
H 0.089176 1.973549 0.297623
H -2.814051 2.824374 0.728271
H 1.473659 -3.078640 1.303271
H 0.646083 -1.686596 -1.198889
H -0.388393 -2.671901 0.415308
H 1.834346 1.557020 -1.603529
H 2.093685 -0.026775 -1.597620
H 1.936843 4.128017 -1.401184
H -2.592579 -2.064464 -2.793013
O -1.345066 -0.021040 1.194146
O -0.167825 -3.400842 1.031561
O -1.417928 0.929070 -1.431928
O 2.333644 -2.595007 1.227048
O 2.667859 -1.734906 -1.388809
O -2.441936 -1.574965 -1.981245
O 1.407541 -0.010293 1.139766
O -1.614838 2.526654 2.165864
O -3.038390 -2.016635 0.650640
O -3.087469 2.627749 -0.182171
O 1.957733 3.290175 -0.931220
O -0.135365 -1.348532 -0.744733
O 1.417190 0.679280 -1.546175
Al -0.002029 0.378989 -0.175839
O 3.420968 1.761315 0.850076
H 3.163490 2.515087 0.294356
H 4.150776 2.043789 1.404414

21.0, 0.7; 30.9, 0.9; 40.3, 0.6; 44.3, 1.8; 58.8, 1.2; 69.2, 2.0; 75.1, 2.1; 83.2, 0.8; 94.2, 5.2; 94.2, 1.7; 100.3, 5.1; 124.3, 19.7; 128.9, 11.6; 146.1, 1.6; 162.7, 6.2; 167.0, 12.2; 172.1, 4.6; 173.9, 7.4; 180.2, 95.3; 199.8, 12.5; 207.6, 59.3; 211.4, 35.6; 218.2, 13.9; 225.9, 1.6; 232.4, 5.8; 241.2, 10.2; 245.3, 8.8; 250.2, 2.8; 259.7, 39.8; 262.7, 36.2; 283.5, 42.4; 293.0, 11.2; 298.8, 2.9; 307.7, 130.6; 314.2, 52.1; 325.4, 43.3; 328.4, 18.0; 332.8, 88.5; 336.4, 20.0; 342.1, 53.1; 348.0, 4.5; 351.5, 9.1; 369.6, 92.2; 375.4, 1.0; 380.0, 80.8; 387.1, 21.6; 398.6, 44.2; 428.8, 124.3; 448.0, 35.2; 461.7, 216.3; 465.2, 25.2; 470.9, 131.2; 476.6, 64.7; 491.8, 155.5; 522.4, 30.8; 534.0, 120.4; 561.8, 193.8; 568.2, 67.1; 597.1, 27.5; 598.9, 170.9; 602.7, 36.9; 626.1, 4.5; 640.0, 42.8; 644.6, 72.5; 679.0, 174.0; 683.0, 145.8; 699.3, 294.9; 713.2, 54.2; 736.1, 343.6; 783.2, 219.8; 800.1, 301.8; 817.9, 50.8; 830.0, 76.1; 873.8, 25.1; 886.9, 238.7; 890.3, 273.2; 934.9, 228.2; 954.9, 115.8; 991.2, 198.2; 1024.9, 227.9; 1047.8, 89.5; 1076.3, 253.6; 1562.3, 71.8; 1565.9, 447.4; 1590.0, 75.8; 1608.4, 164.2; 1610.9, 224.1; 1614.8, 86.3; 1617.9, 5.2; 1625.6, 97.4; 1636.4, 64.9; 1652.8, 107.8; 1661.7, 8.0; 1673.1, 28.0; 1688.9, 170.5; 1704.6, 74.1; 3357.7, 672.6; 3385.4, 332.6; 3421.3,1414.2; 3487.7, 831.6; 3497.2, 546.4; 3543.1, 234.3; 3573.3, 385.7; 3586.6, 938.9; 3609.8, 783.3; 3625.9, 527.4; 3639.1, 936.9; 3648.7, 18.5; 3652.7, 959.4; 3694.1, 337.0; 3703.1, 558.9; 3739.5, 625.6; 3745.1, 568.5; 3840.1, 332.5; 3936.3, 227.5; 3938.4, 149.8; 3941.6, 145.3; 3944.6, 138.6; 3948.9, 192.2; 3950.1, 97.9; 3961.2, 122.3; 3963.5, 191.1; 3967.0, 165.2;

-1312.463066310 -1312.103183 -1312.068738 -1312.1647

11-6c-2
H -0.801410 1.668167 1.683909
H -2.376216 1.433964 1.652803
H -0.640116 1.962594 -1.498910
H -2.232764 1.736216 -1.690159
H -1.893251 -1.819603 1.854987
H -0.376163 -1.977017 1.430320
H 1.782226 -1.273979 1.621598
H 1.781943 -2.754516 2.157322
H 0.222203 -0.004678 -0.028659
H -3.724018 -0.876590 -0.116917
H -0.294039 -1.810029 -1.691888
H -1.846268 -1.835001 -2.050615
O -1.516842 1.001290 1.595875
O -1.455571 1.545647 -1.157318
O -1.285447 -1.769297 1.111394
O 1.354333 -2.170250 1.525147
O -3.131689 -0.133211 -0.173972
O -1.132136 -1.301208 -1.691493
Al -1.376174 -0.121382 -0.066292
O 0.729552 2.531487 1.426626
H 0.873574 2.669935 0.469705
H 0.975184 3.347619 1.871119
H 2.757656 0.310245 0.587690
H 1.922041 0.959592 1.694744
H 1.401678 -2.443958 -0.254490
H 1.748738 -3.155141 -1.600649
O 2.521124 0.216058 1.523800
O 1.359394 -2.356569 -1.232751
H 2.200510 -0.685443 -1.450194
H 3.492441 0.064941 -1.878483
H 1.656556 1.633297 -1.458130
H 1.497958 3.114918 -1.919378
O 2.679289 0.163311 -1.375248
O 1.098216 2.436797 -1.367551

58.2, 4.9; 59.1, 5.4; 72.3, 3.2; 77.6, 0.4; 88.3, 1.2; 96.8, 1.0; 123.4, 8.4; 132.3, 3.5; 150.9, 1.7; 156.2, 9.8; 170.3, 0.7; 174.0, 11.4; 180.9, 12.8; 185.7, 14.9; 193.1, 1.7; 201.9, 3.5; 212.9, 8.8; 219.5, 7.7; 222.9, 20.6; 247.7, 10.6; 257.6, 36.1; 261.3, 6.1; 277.0, 11.2; 285.0, 6.2; 291.4, 18.8; 300.1, 47.4; 306.5, 6.1; 314.7, 56.9; 320.5, 48.9; 338.2, 1.7; 353.9, 113.8; 362.2, 74.5; 367.3, 44.8; 383.7, 18.3; 413.8, 85.8; 421.0, 11.0; 437.0, 168.6; 450.5, 75.6; 471.5, 11.6; 481.1, 44.1; 496.8, 155.2; 506.2, 53.0; 516.2, 56.7; 548.9, 32.3; 562.9, 189.0; 572.0, 77.8; 585.0, 270.1; 594.5, 42.1; 620.3, 78.4; 640.5, 147.0; 657.7, 55.4; 676.4, 36.2; 689.4, 60.9; 724.2, 343.0; 748.5, 180.0; 777.7, 282.3; 800.5, 258.6; 851.3, 78.3; 861.5, 232.2; 899.0, 151.1; 923.6, 160.4; 946.3, 188.5; 961.9, 194.5; 1094.5, 115.4; 1574.2, 106.2; 1602.9, 222.8; 1608.0, 61.3; 1621.9, 145.8; 1630.8, 91.9; 1632.3, 57.0; 1640.5, 17.4; 1642.5, 152.8; 1654.4, 68.6; 1681.4, 34.8; 1911.6, 209.3; 3234.5,1016.1; 3449.7, 239.1; 3507.6, 509.8; 3528.0, 619.1; 3534.0, 969.5; 3571.2, 680.7; 3600.2, 860.2; 3642.2, 567.2; 3650.3, 439.9; 3735.2, 186.5; 3798.2, 535.9; 3887.8, 153.7; 3914.9, 160.5; 3919.3, 71.9; 3922.6, 50.0; 3923.6, 156.3; 3925.3, 240.3; 3926.1, 295.6; 3928.6, 144.8; 3931.5, 79.1; 4044.8, 74.4;

-1083.130671380 -1082.847836 -1082.820934 -1082.898571

11-6c-3
H 2.128750 -0.861184 1.386825
H 1.809683 0.683156 1.405719
H -2.301072 3.155009 0.781658
H 2.240157 -0.432240 -1.373388
H 1.752786 -1.896157 -1.194079
H -3.420440 1.289214 -0.497580
H 4.028492 -1.083702 0.201954
H -4.362058 0.794451 -1.641356
H 1.359470 2.107524 0.046210
H -1.808968 0.682495 1.404608
H -2.127577 -0.861711 1.387997
H -4.115637 -2.339120 1.123111
H -4.026244 -1.081972 0.201863
H 0.000577 -2.173626 0.414817
H 4.113283 -2.342425 1.121407
H 4.360691 0.795567 -1.641017
H 0.000064 1.208887 -1.470198
H -1.359913 2.107013 0.046221
H -1.753108 -1.896729 -1.192208
H -2.240038 -0.433047 -1.374849
H 3.420200 1.289931 -0.496286
H 2.300238 3.156438 0.780678
O 1.401053 -0.201220 1.386639
O -2.236195 2.246241 0.479712
O 1.436924 -0.994711 -1.337374
O 3.504631 -1.739826 0.687339
O -3.731405 0.496417 -0.980398
O 2.235378 2.247147 0.480297
O -1.399910 -0.201709 1.387177
O -3.504796 -1.738818 0.688956
O -0.000202 1.152363 -0.511273
O -1.437002 -0.995599 -1.336908
O 3.731011 0.497205 -0.979273
Al 0.000367 -0.615976 0.066058

40.0, 0.1; 50.8, 4.3; 60.5, 0.2; 66.2, 0.2; 75.4, 3.0; 79.4, 0.0; 117.6, 2.7; 126.7, 0.4; 142.2, 7.0; 152.3, 0.9; 172.0, 9.5; 177.2, 15.2; 190.5, 10.5; 205.6, 2.1; 212.0, 5.7; 218.6, 0.4; 236.3, 20.3; 240.3, 14.9; 267.5, 15.1; 270.0, 16.9; 286.2, 34.3; 289.5, 5.8; 312.8, 39.5; 313.1, 26.5; 319.2, 7.5; 331.2, 73.3; 334.8, 0.1; 338.1, 38.3; 338.7, 0.2; 339.3, 23.0; 350.6, 59.4; 356.3, 40.1; 364.7, 47.2; 385.9, 17.4; 424.4, 136.4; 426.6, 175.1; 431.4, 116.7; 438.4, 18.9; 448.8, 125.8; 453.7, 14.3; 468.2, 8.8; 492.7, 99.1; 518.1, 96.2; 534.4, 13.4; 559.8, 82.2; 594.3, 120.9; 606.3, 42.6; 646.4, 148.4; 661.0, 127.7; 661.8, 332.0; 688.8, 63.0; 733.5, 326.9; 740.4, 81.2; 766.7, 685.2; 794.6, 0.6; 820.4, 7.0; 855.3, 119.2; 880.7, 709.9; 912.9, 36.6; 935.6, 24.2; 939.1, 79.9; 951.7, 305.9; 985.9, 135.6; 1019.2, 18.7; 1572.4, 13.8; 1581.0, 345.0; 1582.2, 9.1; 1604.5, 213.4; 1624.5, 0.2; 1628.5, 33.5; 1670.1, 187.0; 1676.1, 10.6; 1683.7, 171.9; 1692.3, 8.1; 1897.6, 203.2; 3393.0,1089.7; 3443.0, 118.4; 3525.7, 524.0; 3536.3, 580.9; 3537.1,1030.7; 3548.4, 360.5; 3603.9, 890.9; 3607.7, 235.6; 3681.1, 24.7; 3688.9,1381.9; 3758.9, 152.9; 3759.9, 382.5; 3856.7, 0.3; 3858.3, 314.6; 3926.3, 54.4; 3932.3, 123.1; 3932.6, 227.3; 3944.3, 54.6; 3947.0, 270.3; 3952.7, 45.5; 3953.0, 295.9;

-1083.139585490 -1082.855378 -1082.828975 -1082.906470

11-6c-4
H 2.590951 -3.016643 0.321779
H 2.856180 -1.880562 1.380525
H -0.456194 -0.255598 3.763790
H 2.666320 0.937163 -1.049349
H 2.793695 -0.249837 -2.083053
H -1.781350 0.832806 2.022363
H -2.961090 -0.092475 0.265989
H -3.002090 1.783537 1.837297
H -1.074884 3.448282 -0.748662
H -0.677888 -1.499236 1.525413
H -1.419374 -1.707973 0.174087
H -2.142021 -1.345730 -3.500977
H -2.702752 -0.936303 -2.100013
H 1.018897 -2.036526 -1.510678
H -3.883324 -1.335922 0.118100
H 2.766852 3.288315 -0.213762
H 1.742603 0.948640 0.997655
H 0.199276 0.068232 2.333997
H -0.754042 -0.075176 -2.028879
H -0.352921 1.173202 -1.084631
H 1.288811 2.791528 -0.272177
H -1.185534 2.397898 0.396542
O 2.230987 -2.226311 0.736068
O -0.641218 -0.176117 2.825823
O 2.659650 -0.038046 -1.154153
O -3.067323 -0.943350 -0.204208
O -2.322864 1.309920 1.347937
O -0.591752 2.749528 -0.298923
O -0.543811 -1.782713 0.599943
O -2.099543 -0.677566 -2.814019

O 1.181817 0.172410 1.072225
O -0.121627 0.262139 -1.348843
O 2.219423 2.502999 -0.285742
Al 1.018459 -0.958521 -0.334938

40.5, 2.5; 48.2, 1.9; 55.0, 1.6; 69.5, 1.5; 70.9, 3.7; 94.6, 0.7; 98.5, 1.9; 131.0, 3.1; 133.0, 4.4; 139.9, 2.5; 167.0, 1.3; 175.9, 26.5; 185.0, 3.0; 189.7, 11.9; 206.1, 6.8; 208.2, 12.7; 218.3, 12.6; 225.5, 95.7; 238.5, 70.5; 246.4, 26.9; 248.7, 21.6; 259.6, 63.5; 267.6, 33.6; 277.7, 40.9; 289.7, 2.4; 298.9, 7.5; 305.0, 44.1; 307.6, 24.9; 316.1, 6.6; 335.2, 21.9; 337.7, 24.4; 366.5, 13.2; 373.9, 35.4; 386.7, 69.1; 395.5, 38.3; 403.3, 110.5; 417.8, 31.1; 435.6, 25.8; 442.5, 269.5; 462.0, 42.0; 479.0, 71.2; 493.4, 33.4; 497.1, 49.1; 522.0, 171.0; 542.4, 111.3; 547.6, 16.1; 573.7, 79.3; 587.1, 51.7; 613.1, 118.3; 623.6, 129.0; 675.3, 193.8; 703.0, 249.6; 738.8, 153.8; 768.7, 166.6; 800.5, 74.6; 819.8, 74.1; 855.9, 444.5; 874.2, 459.0; 889.8, 47.2; 900.4, 24.0; 920.3, 342.2; 956.5, 178.2; 967.3, 108.5; 1115.8, 109.4; 1585.9, 203.5; 1588.6, 63.2; 1595.1, 47.2; 1600.9, 91.7; 1630.7, 45.8; 1636.6, 130.6; 1647.8, 137.7; 1659.4, 52.9; 1665.7, 41.3; 1692.6, 96.6; 1899.9, 235.9; 3112.3,1025.6; 3407.4, 974.8; 3427.5, 707.1; 3552.3, 356.1; 3555.2, 252.3; 3613.3,1163.8; 3622.4, 676.7; 3632.0, 309.5; 3667.8,1141.7; 3692.7, 432.3; 3754.2, 265.7; 3846.9, 65.3; 3908.2, 207.3; 3914.1, 207.9; 3915.7, 116.6; 3927.7, 197.9; 3930.8, 123.8; 3948.4, 139.9; 3949.1, 123.4; 3964.1, 166.3; 3967.0, 185.6;

-1083.134063460 -1082.851919 -1082.824676 -1082.903995

11-6c-5
H -1.827828 0.091453 -1.934742
H -1.634561 1.140953 -0.829071
H 1.078918 3.227463 -0.706811
H -1.834403 -0.956279 1.381851
H -2.485631 -1.449874 0.033888
H 3.492856 -0.184081 0.718659
H 4.411096 1.335037 -1.036837
H 3.663059 -0.891375 2.101427
H -0.751125 0.662358 1.924991
H 2.352869 0.069782 -1.447431
H 1.618609 -0.865971 -2.515291
H -2.666607 1.515020 1.153383
H -3.329504 2.626834 0.276603
H -0.482246 -1.964108 -1.672233
H 3.011746 1.813731 -0.482381
H -4.526342 -0.986343 -1.420364
H 1.240327 0.077586 1.223868
H 0.957938 1.900846 0.128554
H 1.075814 -3.203717 0.106031
H 1.912904 -2.032508 0.794296
H -3.913456 0.214942 -0.633112
H -1.571491 0.796454 3.278435
O -1.139058 0.516736 -1.400454
O 1.560458 2.630354 -0.132198
O -1.659388 -1.448580 0.557783
O 3.584995 1.026051 -0.656396
O 3.072493 -0.801263 1.349563
O -1.652751 0.643930 2.334197
O 1.565724 -0.488371 -1.633577
O -2.979296 1.732569 0.249590
O 0.440708 0.294838 0.727832
O 1.032506 -2.312618 0.463804
O -3.706365 -0.625801 -1.073318
Al -0.057934 -0.944689 -0.518333

34.4, 0.6; 45.6, 11.1; 60.4, 1.9; 62.4, 7.3; 85.6, 0.8; 90.5, 0.7; 118.0, 2.2; 122.7, 4.4; 135.1, 2.7; 145.0, 3.6; 171.2, 22.4; 178.2, 9.7; 192.4, 9.5; 204.5, 17.2; 208.1, 31.4; 216.9, 11.8; 224.3, 63.1; 232.6, 1.9; 246.8, 26.9; 254.3, 37.7; 271.1, 40.0; 275.4, 16.0; 293.0, 8.3; 295.2, 11.2; 308.0, 7.2; 319.4, 31.4; 325.6, 4.5; 327.7, 118.6; 340.0, 21.6; 341.2, 5.5; 353.0, 11.6; 362.8, 39.3; 373.0, 6.6; 376.1, 65.5; 408.7, 25.6; 418.3, 58.9; 423.3, 88.3; 438.2, 229.0; 453.5, 74.6; 466.4, 151.5; 475.3, 25.8; 484.3, 63.2; 528.3, 10.1; 557.4, 129.8; 584.3, 60.7; 591.0, 50.9; 602.0, 151.7; 623.0, 41.4; 663.1, 89.0; 672.8, 48.1; 696.1, 272.9; 719.4, 92.5; 749.5, 427.7; 762.8, 158.5; 778.8, 361.7; 818.2, 47.8; 845.3, 119.6; 873.1, 42.2; 891.4, 468.8; 911.5, 200.6; 921.1, 146.3; 950.5, 164.4; 988.7, 44.6; 1021.4, 202.5; 1585.6, 225.5; 1594.5, 80.6; 1606.3, 153.5; 1612.9, 34.9; 1627.6, 4.1; 1632.0, 125.3; 1655.1, 68.2; 1657.7, 103.1; 1677.0, 6.1; 1690.3, 194.3; 1893.0, 228.3; 3362.9,1065.4; 3380.3, 301.5; 3500.5, 177.4; 3536.6, 545.7; 3543.9, 492.9; 3553.1,1518.8; 3586.9, 88.6; 3606.5, 857.1; 3633.5, 552.2; 3672.1, 738.8; 3732.7, 274.0; 3792.1, 303.9; 3830.8, 320.2; 3925.3, 138.0; 3931.4, 204.1; 3933.8, 152.7; 3935.8, 203.6; 3942.7, 105.7; 3943.8, 215.9; 3945.8, 132.6; 3964.9, 126.4;

-1083.138278910 -1082.854885 -1082.828242 -1082.906353

References

[1] a) A. Akhgarnusch, W. K. Tang, H. Zhang, C.-K. Siu, M. K. Beyer, *Phys. Chem. Chem. Phys.* **2016**, *18*, 23528; b) M. Allemann, H. Kellerhals, K. P. Wanczek, *Int. J. Mass Spectrom. Ion Process.* **1983**, *46*, 139; c) C. Berg, T. Schindler, G. Niedner-Schatteburg, V. E. Bondybey, *J. Chem. Phys.* **1995**, *102*, 4870; d) A. Akhgarnusch, R. F. Höckendorf, M. K. Beyer, *J. Phys. Chem. A* **2015**, *119*, 9978.

[2] P. Caravatti, M. Allemann, *Org. Mass Spectrom.* **1991**, *26*, 514.

[3] a) V. E. Bondybey, J. H. English, *J. Chem. Phys.* **1981**, *74*, 6978; b) T. G. Dietz, M. A. Duncan, D. E. Powers, R. E. Smalley, *J. Chem. Phys.* **1981**, *74*, 6511.

[4] A. G. Marshall, C. L. Hendrickson, G. S. Jackson, *Mass Spectrom. Rev.* **1998**, *17*, 1.

[5] a) R. L. Wong, K. Paech, E. R. Williams, *Int. J. Mass Spectrom.* **2004**, *232*, 59; b) O. P. Balaj, C. B. Berg, S. J. Reitmeier, V. E. Bondybey, M. K. Beyer, *Int. J. Mass Spectrom.* **2009**, *279*, 5.

[6] a) D. Thölmann, D. S. Tonner, T. B. McMahon, *J. Phys. Chem.* **1994**, *98*, 2002; b) R. C. Dunbar, *Mass Spectrom. Rev.* **2004**, *23*, 127; c) T. Schindler, C. Berg, G. Niedner-Schatteburg, V. E. Bondybey, *Chem. Phys. Lett.* **1996**, *250*, 301; d) P. D. Schnier, W. D. Price, R. A. Jockusch, E. R. Williams, *J. Am. Chem. Soc.* **1996**, *118*, 7178; e) M. Sena, J. M. Riveros, *Rapid Commun. Mass Spectrom.* **1994**, *8*, 1031.

[7] B. S. Fox, M. K. Beyer, V. E. Bondybey, *J. Phys. Chem. A* **2001**, *105*, 6386.
